# The effect of cartilage decellularized extracellular matrix-chitosan compound on treating knee osteoarthritis in rats

**DOI:** 10.7717/peerj.12188

**Published:** 2021-10-12

**Authors:** Deng Chen, Yaxin Zhang, Qun Lin, Duoyun Chen, Xiaolei Li, Jihang Dai, Yu Sun

**Affiliations:** 1 Department of Orthopedics, Northern Jiangsu People’s Hospital Affiliated to Yangzhou University, Yangzhou, Jiangsu, China; 2 Dalian Medical University, Dalian, Liaoning, China

**Keywords:** dECM, Articular cartilage, Osteoarthritis, Chitosan, Compound

## Abstract

Knee osteoarthritis (KOA) refers to a common disease in orthopaedics, whereas effective treatments have been rarely developed. As indicated from existing studies, chondrocyte death, extracellular matrix degradation and subchondral bone injury are recognized as the pathological basis of KOA. The present study aimed to determine the therapeutic effect of decellularized extracellular matrix-chitosan (dECM-CS) compound on KOA. In this study, rat knee cartilage was decellularized, and a satisfactory decellularized extracellular matrix was developed. As suggested from the *in vitro* experiments, the rat chondrocytes co-cultured with allogeneic dECM grew effectively. According to the results of the alamar blue detection, dECM did not adversely affect the viability of rat chondrocytes, and dECM could up-regulate the genes related to the cartilage synthesis and metabolism. As reported from the animal experiments, dECM-CS compound could protect cartilage, alleviate knee joint pain in rats, significantly delay the progress of KOA in rats, and achieve high drug safety. In brief, dECM-CS compound shows a good therapeutic effect on KOA.

## Introduction

KOA refers to a degenerative joint disease in orthopaedics. It is primarily characterized by synovitis, degeneration of cartilage, formation of osteophyte, as well as sclerosis of subchondral bone (Mandl, 2019). The main clinical treatment complies with the mitigation of pain symptoms at the early stage of this disease. When osteoarthritis of the knee develops to advanced stage, knee replacement is recognized as the main existing treatment (Bannuru et al., 2019; Meiyappan et al., 2020; Bijlsma, Berenbaum & Lafeber, 2011). Thus far, no effective drug is capable of preventing the progression of KOA. The incidence and prevalence of KOA will further rise over the coming decades as impacted by the aging population, the rising obesity rates and the high rates of traumatic knee injuries (Martel-Pelletier et al., 2016). This is recognized as a public health crisis, and rigorous high-quality KOA clinical research is urgently required to ensure patients receive safe and effective treatments.

One of the OA progression mechanism is a reduction of chondrocyte numbers and ECM degradation in cartilage (Carballo et al., 2017). Chondrocytes are unique cells in articular cartilage, which are capable of synthesizing ECM components. ECM largely comprises COL2 and GAGs, which are of a critical significance for the biomechanical properties of cartilage. It protects the articular surface of the bone from abrasion, distributes the applied loads over a larger joint area, and creates a smooth, lubricated surface for the joint movement to reduce friction (Ravindran et al., 2015). dECM materials have been extensively employed in a wide range of biological and medical fields. The ECM obtained by decellularization exhibits low immunogenicity and retains the characteristics of ECM (Lee et al., 2020; Yao et al., 2019). Chitosan (CS) is a non-toxic natural polymer, which has exhibits biodegradability and biocompatibility; it acts as a prominent bio-material (Comblain et al., 2017). By mixing chitosan with other natural or synthetic polymers, the multifaceted performance of the mentioned biological scaffolds can be effectively promoted (*e.g*., controlling the porosity and the water retention, reducing their biodegradation, enhancing their bioactivity and biocompatibility, and improving their mechanical properties) (Wang et al., 2020). The CHI/SF/ESM hydrogels with ECM-mimicking interconnected structures were successfully synthesized by Terin Adal etc. These hydrogels show superior mechanical properties for desired application and support growth, adhesion and differentiation of Human chondrocyte cells where high viability was observed under normal *in vitro* cell culturing conditions (Adali, Kalkan & Karimizarandi, 2019).

Most of the mentioned biological composite scaffolds made by CS exhibit no cytotoxicity and can promote the cell attachment and proliferation for the cartilage repair (Kean & Thanou, 2010).

**Figure 1:**
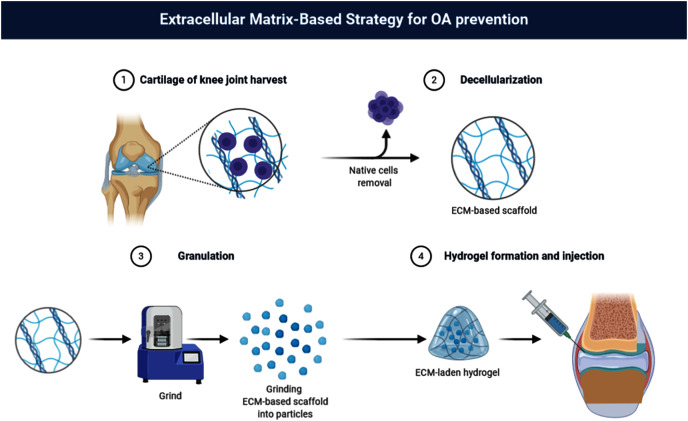
Flow chart. Flow chart of dECM-CS compound preparation (picture is not copyrighted). Images adapted from BioRender Basic plan (https://app.biorender.com/).

The author has previously shown that the concentration of 40% (w/v) dECM suspension is the optimal in the rabbit KOA therapy (Zhang et al., 2020). In the present study, dECM of rat cartilage and chitosan was used to create dECM-CS compound to determine the therapeutic effect of dECM-CS compound to treat KOA. Furthermore, the experiments *in vitro* were continuously performed to explore the intervention effect of dECM on chondrocytes (Fig. 1).

## Materials and Methods

### Decellularization

In this study, the cartilage pieces were obtained from the femoral side articular surface of the fresh rat knee joints. Subsequently, the specimens were washed completely with phosphate buffered saline (PBS) to ensure that the specimen was free of impurities. The method adopted complied with the description reported by Kheir et al. (2011). Such a method was adopted in this experiment based on some improvements. In brief, the fresh rat knee cartilage underwent several freeze-drying cycles. Subsequently, the cartilage was treated with aprotinin, SDS, EDTA and tris-Hcl, then with the DNase and RNase reaction, and lastly washed with TritonX-100. To achieve the disinfection, Penicillin (100 U/ml), streptomycin (100 μg/ml), and fluconazole (2.5 μg/ml) were added to all the solutions used.

### Decellularization evaluation

#### Histology

First, some fresh undealted rat cartilage pieces were obtained, and the cartilage pieces were decellularized. The specimen was fixed with paraformaldehyde (4% [w/v]) at normal temperatures for 24 h. Next, those tissues were decalcified with EDTA solution (25% [w/v], pH 7.0) for 4–5 weeks till the hardness of cartilage tissue was exactly right to be sectioned. In this period, the EDTA solution was changed once per three days. Lastly, the hematoxylin-eosin (HE) staining, the alcian blue staining and the scanning electronic microscope (SEM) were used to identify whether the decellularization was successful.

#### Biochemical analysis of GAG and DNA content

The fresh or dealted cartilage pieces were placed in a papain solution to be digested at 60 °C for 12 h. Next, this reaction solution was centrifuged at 10,000 *g* for 30 min. The GAG content was evaluated by employing the dimethylmethylene blue colorimetric quantitative detection kit. By complying with the instructions, a GAG standard curve was plotted by using the standard samples. Subsequently, the exact GAG content was obtained by using such a standard curve. The DNA content was determined by employing the dsDNA HS Assay Kit for Qubit. Likewise, a DNA standard curve was plotted by using the standard samples, Afterwards, the exact DNA content was obtained. The content of GAG or DNA was the wet weight per milligram of the sample, as expressed in micrograms.

#### Scanning electron microscopy (SEM)

The fresh and decellularized rat cartilage pieces were immersed in a glutaraldehyde solution (2.5% [w/v]) for 2 h. Next, the tissue was flushed with a sodium dimethylarsenate buffer (pH 7.4). Afterwards, the pieces were fixed with osmium tetroxide (1% [w/v]) and sprayed gold on the tissue for 30 s by the ion sputtering apparatusthe. Lastly, the samples were scanned with the SEM.

### Manufacturing of dECMs-CS compound, dECMs suspension

After the decellularization, a tissue lapping apparatus (Servicebio, Wuhan, China) was adopted to grind the decellularized cartilage piece particle. Subsequently, the dECM particle was washed intensively with normal saline. So dECM is a non-immunogenic biological tissue material obtained from rat knee cartilage tissue through decellularized process, and its main component is GAGs. Finally, the dECMs were mixed with chitosan liquid or normal saline, respectively, at a concentration of 40% [w/v].

### Measuring the dECM particle

The dECM particles were stained with alcian blue, and then the particles were measured with imageJ.

### Identification of chondrocytes

The collagen II immunofluorescence staining and the alcian blue staining were used to identify the cytology.

### Rat chondrocytes cultured with dECM

The rat chondrocytes were cultured in a medium supplemented by 40% dECM suspension for 3–5 days to observe their growth status.

### Alamar blue detection

The dECM was used to interfere with rat chondrocytes, and alarmarBlue™Cell Viability Assay Reagent (Solarbio, Beijing, China) was introduced to determine the chondrocytes viability. In brief, chondrocytes were seeded in 96-well plates at a concentration of 40,000 cells/ml, and 40% dECM was adopted to interfere with the chondrocytes for 1 d, 3 d, 5 d and 7 d. Subsequently, 10 μl alamar blue reagent was added into the respective well, and the mentioned well was placed in the cell incubator for 4 h (37 °C, 5% CO_2_). The reduction rate of alamar blue reagent was calculated by complying with the manual.

### Reverse transcription polymerase chain reaction (RT-PCR)

The primer sequences are listed in Table 1. Based on the instructions, the total RNAs were extract by treating the chondrocytes of rats with the TRIzol reagent (Sigma, Ronkonkoma, NY, USA). Next, the cDNA was synthesized by using the Prime Script™ RT Master Mix Kit (Takara, Tokyo, Japan). With the GAPDH as the internal reference control, RT-PCR was performed with the TB Green™ Premix Ex TaqTM II Kit (Takara, Tokyo, Japan). The result was calculated by the instructions. Information regarding the primer sequences is given in Table 1.

**Table 1:** Primers used for RT-qPCR.

Genes	Primer sequence (5′–3′)
Col2A1	S: GCCTCGCGGTGAGCCATGATC
	AS: CTCCATCTCTGCCACGGGGT
ACAN	S: TAGAGGATGTGAGTGGTCTT
	AS: TCCACTAAGGTACTGTCCAC
SOX-9	S: GAGCTGAGCAGCGACGTCATCT
	AS: GGCGGCGCCTGCTGCTTGGACA
PRG4	S: AACAGGGAAGATAGTGGC
	AS: CGTAGTAATCATAGCCGTCA
GAPDH	S: CACTGTGCCCATCTACGA
	AS: TGATGTCACGCACGATTT

**Note:**

S, sense; AS, antisence.

### Establishment of OA model and intra-articular injection treatment

Specific to the *in vivo* experiment, sixty adults, Sprague–Dawley, male, rats weighing 260 ± 10 g were employed for this experiment. We obtained experimental rats from Yangzhou University, and all the rats were raised in the Comparative Medical Center of Yangzhou University. The feeding conditions were quiet, ventilated, clean and dry, the temperature was suitable, feed and drinking water were added regularly. Yangzhou University provided full approval for this research (YZUNSFC2020-LCYXY-24), and the experiment complied with the Guide for the Care and Use of Laboratory Animals (National Academies, 2011). The rats were anesthetized with 7% [w/v] chloral hydrate (0.5 ml/100 g) based on the intraperitoneal injection. A post-traumatic OA model was built through the anterior cruciate ligament transection (ACLT) (Aizah, Chong & Kamarul, 2019; Cohen-Solal, Funck-Brentano & Hay, 2013; Florea et al., 2015). In brief, under general anesthesia, the anterior cruciate ligament of the right knee was transected. In addition, a sham operation was performed on the contralateral knee, while no ligament transection was performed. The rats randomly fell to six groups, *i.e*., normal, sham-operated, ACLT-operated treated with physiological saline, and treated with 100 μl of chitosan, 100 μl of 40% [w/v] dECM-suspension, 100 μl of 40% [w/v] dECM-CS compound, respectively. To prevent the infection, cefazolin was injected intramuscularly 30 min before and three days after the surgery, and the celecoxib was given 1 week after surgery to relieve pain. The rats underwent an intra-articular injection therapy twice a week for 4 or 8 w. At the end of the experiment, all rats were killed with excessive anesthetic drugs to obtain knee joint specimens, and the animal carcasses were uniformly disposed of by the Animal specimen processing Center of Yangzhou University.

### Hot plate test

Pain refers to a prominent clinical manifestation of KOA, and the hot plate test has been extensively described as a good tool to assess the pain of animal models (Barrot, 2012). In this study, the hot plate test was performed to record the response time of rats to pain, and the therapeutic effect of KOA in the rats here was indirectly assessed. A hot plate (Softmaze, Shanghai, China) was employed at a pre-set plate temperature of 52.5 °C as recommended for rats. Moreover, a cut-off time was set to 60 s. The time from when the rat was placed on the hot plate till the first sign of discomfort from the thermal stimulus was recorded immediately. To be specific, the licking, shaking, or stepping of the paws was observed. None showed the signs of thermally-induced damage to the paws once either study was conducted. The observers were blinded to the experiment.

### Weight-bearing asymmetry test

To assess the pain and inflammation of the hind limbs of rats, an incapacitance tester (RWD Biotechnology, Shenzhen, China) was employed to measure the weight distribution of hind limbs (Hamilton et al., 2015). In the static load test, the rats were placed in a retainer, in which the animal could be comfortably maintained, while its rear paws were shelved on two separated sensor plates. By regulating the weight distribution on the rear paw, the animal stood, and the pain was alleviated naturally. The duration was set to 9 s, and the instrument readings indicated the different weight value of the sham (left) hind limb and the ACLT (right) hind limb. The observers were blinded to the experiment.

### Histological analysis

The rat knee joints were obtained, and the specimens were placed in 4% [w/v] paraformaldehyde for 24 h. Subsequently, the specimens were incubated in the EDTA solution (decalcifying solution consisting of 25% [v/w] EDTA, pH 7.0) at ambient temperatures. The EDTA solution was changed per three days. When the specimens were sufficiently soft to be sectioned, they were embedded in paraffin and then sliced. Next, the mentioned histological sections were stained with HE, the safranin O-fast green staining and alcian blue. Furthermore, the Osteoarthritis Research Society International (OARSI) grading system was adopted to score histopathologic variations in osteoarthritic cartilage (Pritzker et al., 2006).

### Identification of hepatorenal toxicity

At 4 weeks and 8 weeks after the knee joint injection, the rats were randomly selected in the respective group. Their liver and kidney tissues were taken, and HE staining sections were performed to observe whether their liver and kidney cell structures were damaged, as well as determine the hepatorenal toxicity of dECM on the rats.

## Results

### Decellularization evaluation

#### Histological

**Figure 2:**
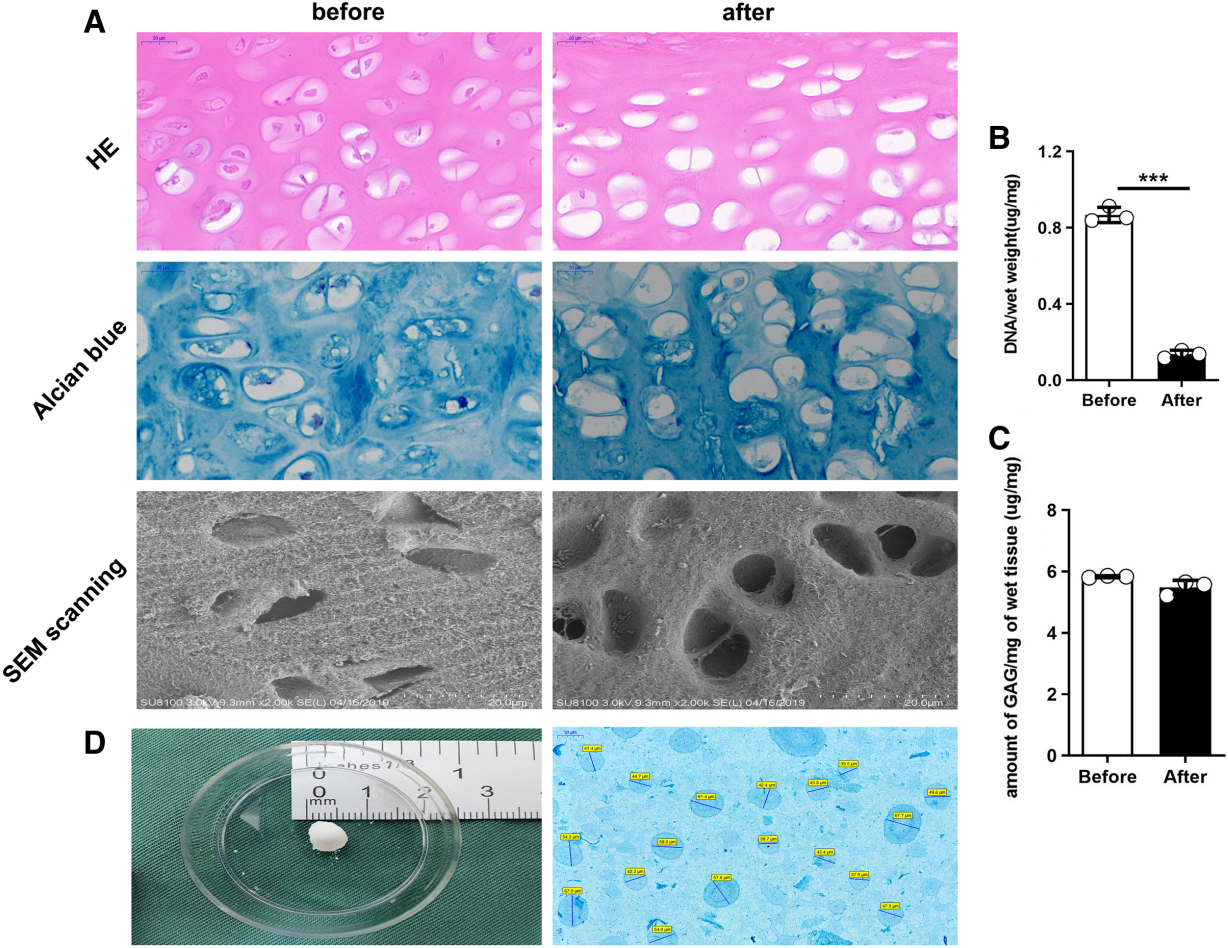
Comparison of rat cartilage tissue before and after the decellularization. (A) HE staining, alcian blue staining and SEM scanning. (B) DNA content. ***P < 0.001. (C) GAG content. (D) Cartilage tissue of rat knee joint, dECM particle characterization alcian blue.

The following figures present two groups of the cartilage tissue by using different histological staining processes. In the fresh undealt cartilage tissue, the HE staining showed the chondrocytes to be embedded in lacunae in the matrix. In the dealt pieces after the decellularization, however, almost all of the chondrocytes were removed away from the tissue. As demonstrated from the figure of the alcian blue staining, the staining intensity between the two group of cartilage pieces was consistent, indicating that GAG was not much to lose (Fig. 2A).

#### Biochemical analysis

The results revealed that before and after the decellularization, the content of GAG per miligram of wet cartilage tissue was 5.83 ± 0.03 μg/mg and 5.48 ± 0.23 μg/mg, and the content of DNA per miligram of wet cartilage tissue was 0.87 ± 0.40 μg/mg and 0.14 ± 0.02 μg/mg. Moreover, no statistically significant difference was reported in GAG content (*t* test, *n* = 3, *P* > 0.05), (Fig. 2C) and a highly significant reduction was identified in the DNA content (*t* test, *n* = 3, *P* < 0.05) (Fig. 2B).

#### Microstructural

As demonstrated from the SEM scanning graph, the dealt cartilage lost the most of cells, the surface structure became loose, the chondrocytes were removed after the decellularization, and almost no cells in the pore after the decellularization (Fig. 2A).

### Staining and measurement of dECM particle

The dECM particle was stained with alcian blue, and the its diameter was measured with Image J. It was indicated that the particle size was 49.38 ± 9.92 μm (Fig. 2D).

### Chondrocytes identification

**Figure 3:**
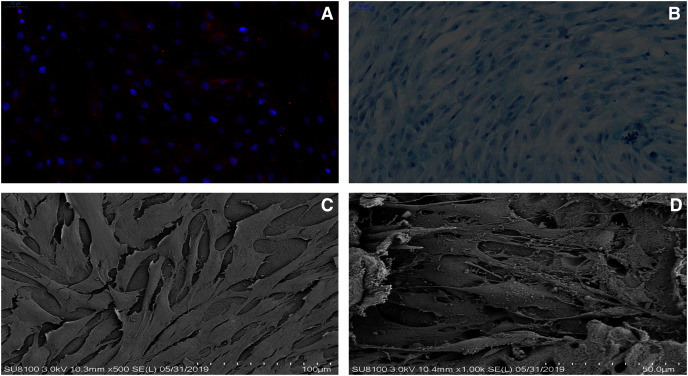
dECM enhance the anabolism of chondrocytes. (A) The rat chondrocytes of knee joint were stained with type II collagen immunofluorescence staining. (B) The rat cartilage of knee joint was stained with alcian blue. (C) The growth state of rat chondrocytes on dECM (original magnification ⊆ 500). (D) The growth state of rat chondrocytes on dECM (original magnification ⊆ 1,000).

The immunofluorescence staining and the alcian blue staining were used to identify the chondrocytes harvest from the rat knee joint. Based on the alcian blue staining, the proteoglycan in the cytoplasm of chondrocytes was stained blue, which revealed that the primary rat chondrocytes were fusiform (Fig. 3B). In addition, type II collagen in the cytoplasm of rat chondrocytes could be stained red through the immunofluorescence (Fig. 3A). As suggested from the results of the two staining methods, the cells extracted from the articular cartilage were chondrocytes.

### The effect of dECM on rat chondrocytes viability, toxicity

**Figure 4:**
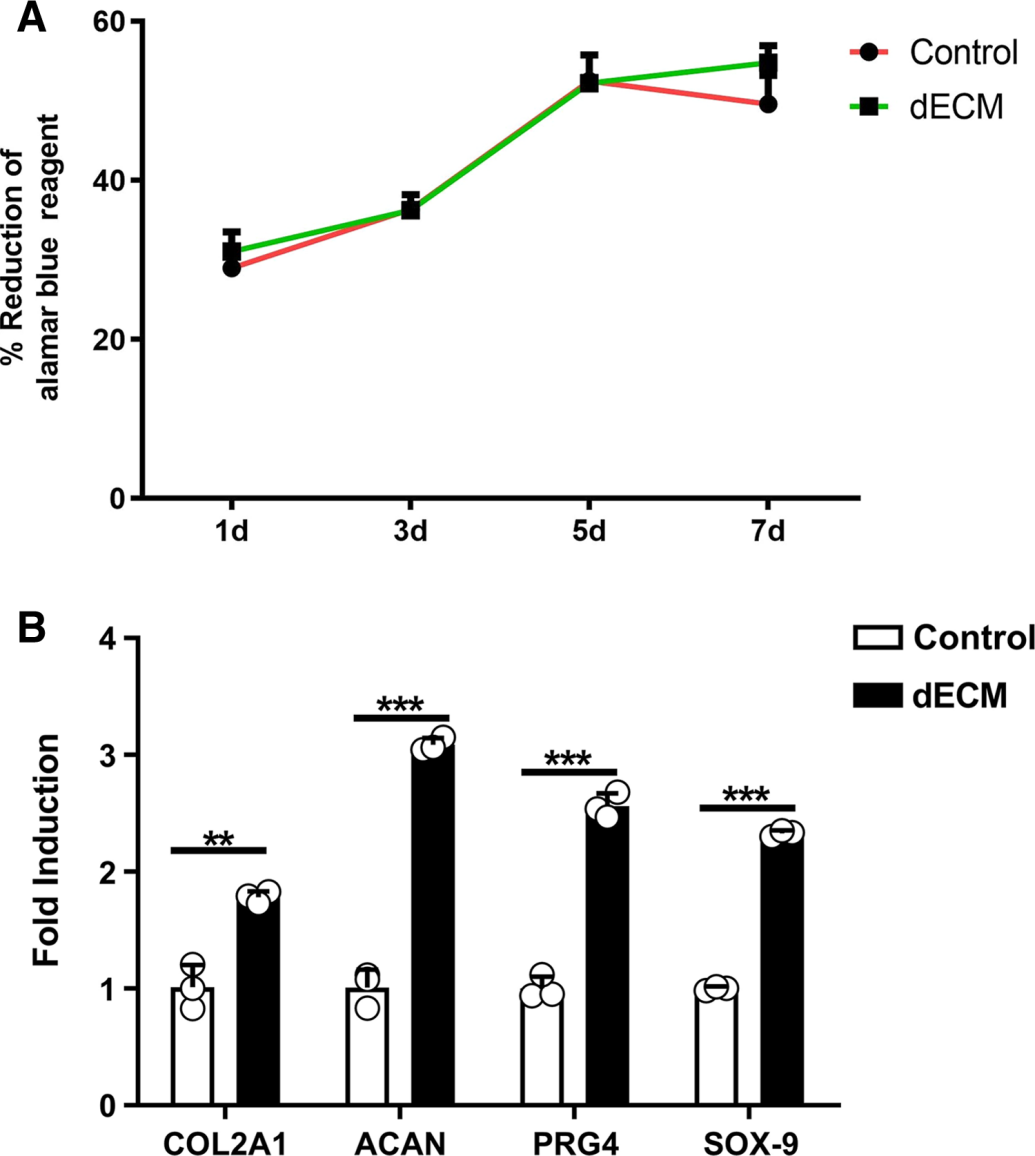
dECM enhance the anabolism of chondrocyteser. (A) Alamar blue assay was performed to verify the viability of rat chondrocytes at 1 d, 3 d, 5 d, and 7 d after the dECM intervention (*n* = 3 for each group). (B) The mRNA levels anabolic genes in rat chondrocytes analyzed by RT-PCR after dECM treatment for 24 h. (*n* = 3 for each group, **P < 0.01, ***P < 0.001).

As indicated by the different magnification electron microscopy, the chondrocytes in the dECM of the knee joint showed the significant growth (Figs. 3C and 3D). According to Alamar Blue staining, the number of chondrocytes in the knee joint of rats in both groups was elevated logarithmically after 3–5 days, and the dECM group achieved more active chondrocyte growth after 7 days (Fig. 4A).

### The expression levels cartilage related genes

Whether dECM up-regulated the expressions of extracellular matrix related genes in rat chondrocytes was investigated. The RT-PCR technology was adopted to indicate that the expressions of cartilage related genes were up-regulated after the dECM intervention. This study observed that the levels of Col2A1, ACAN, PRG4 and Sox9 mRNA increased through the dECM treatment (Fig. 4B).

### Therapeutic effect of dECM-CS compound on KOA in rats

**Figure 5:**
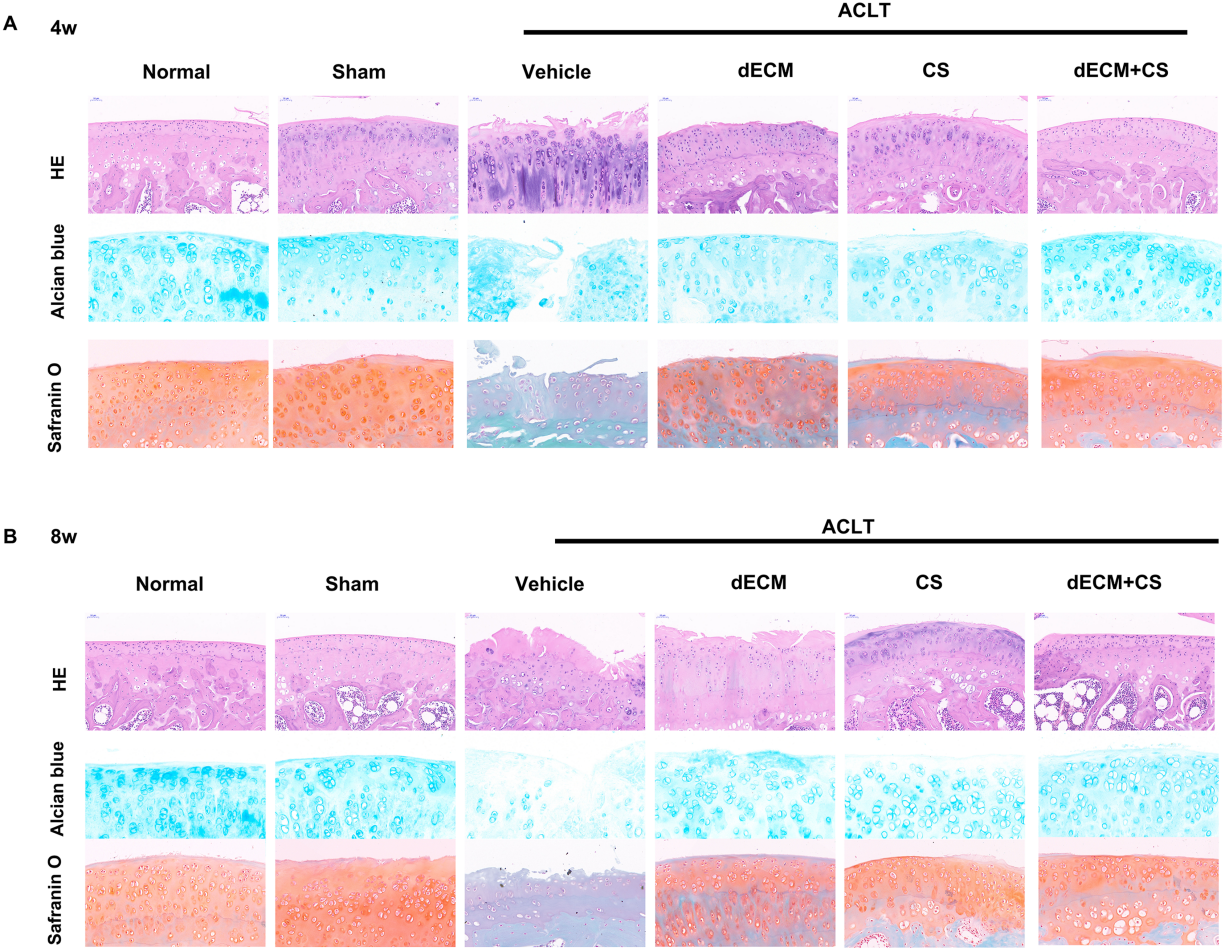
Histological evaluation after intra-articular injection treatment. (A and B) Representative pictures of HE, safranin O-fast green and alcian blue from rats in normal, sham, vehicle, dECM suspension, CS, and dECM-CS compound groups at 4 w and 8 w (*n* = 6 for each group).

**Figure 6:**
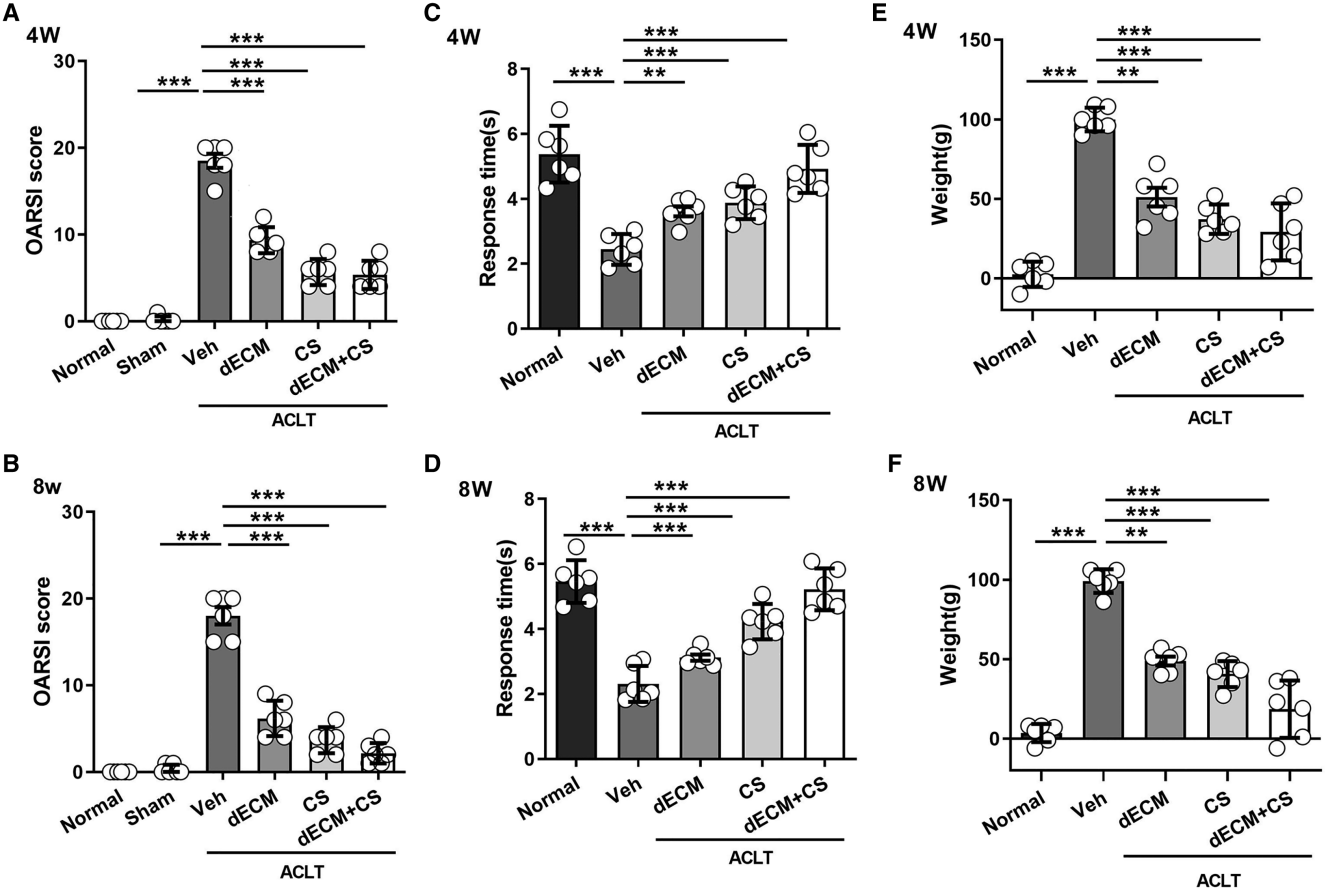
Evaluation after intra-articular injection treatment. (A and B) OARSI scores from rats in the normal, the sham, the vehicle, and the dECM suspension, the CS, and the dECM-CS compound groups at 4 w and 8 w (*n* = 6 for each group). (C and D) Pain response times of rats in the respective group exposed to thermal stimulus at 4 w and 8 w after the treatment (*n* = 6 for each group). (E and F) Weight difference of bilateral lower limbs of rats in the respective group after 4 w and 8 w of treatment (*n* = 6 for each group). All data were expressed as mean ± standard deviation. *P < 0.05, **P < 0.01, ***P < 0.001.

All experimental rats survived after operation and there were no complications such as wound infection. As indicated from the result, the dECM-CS compound treatment significantly delayed the progression of KOA in rats. As suggested from the figure, the articular cartilage erosion and the reduced proteoglycan loss were notably inhibited, which was assessed by using HE, the safranin O-fast green staining green and the alcian blue staining at 4 w or 8 w (Figs. 5A and 5B). This result was further objectively confirmed based on the results of OARSI scores (Figs. 6A and 6B). In comparison with the ACLT control group, the dECM-CS compound treatment group had the significantly lower scores of ACLT rats. By performing the hot-plate test (Figs. 6C and 6D) and the weight-bearing test (Figs. 6E and 6F), it was obviously found that the local injection treatment of dECM-CS compound in ACLT rats could significantly alleviate OA-induced pain after 4 w or 8 w of the treatment.

### dECM hepatorenal toxicity in rats

**Figure 7:**
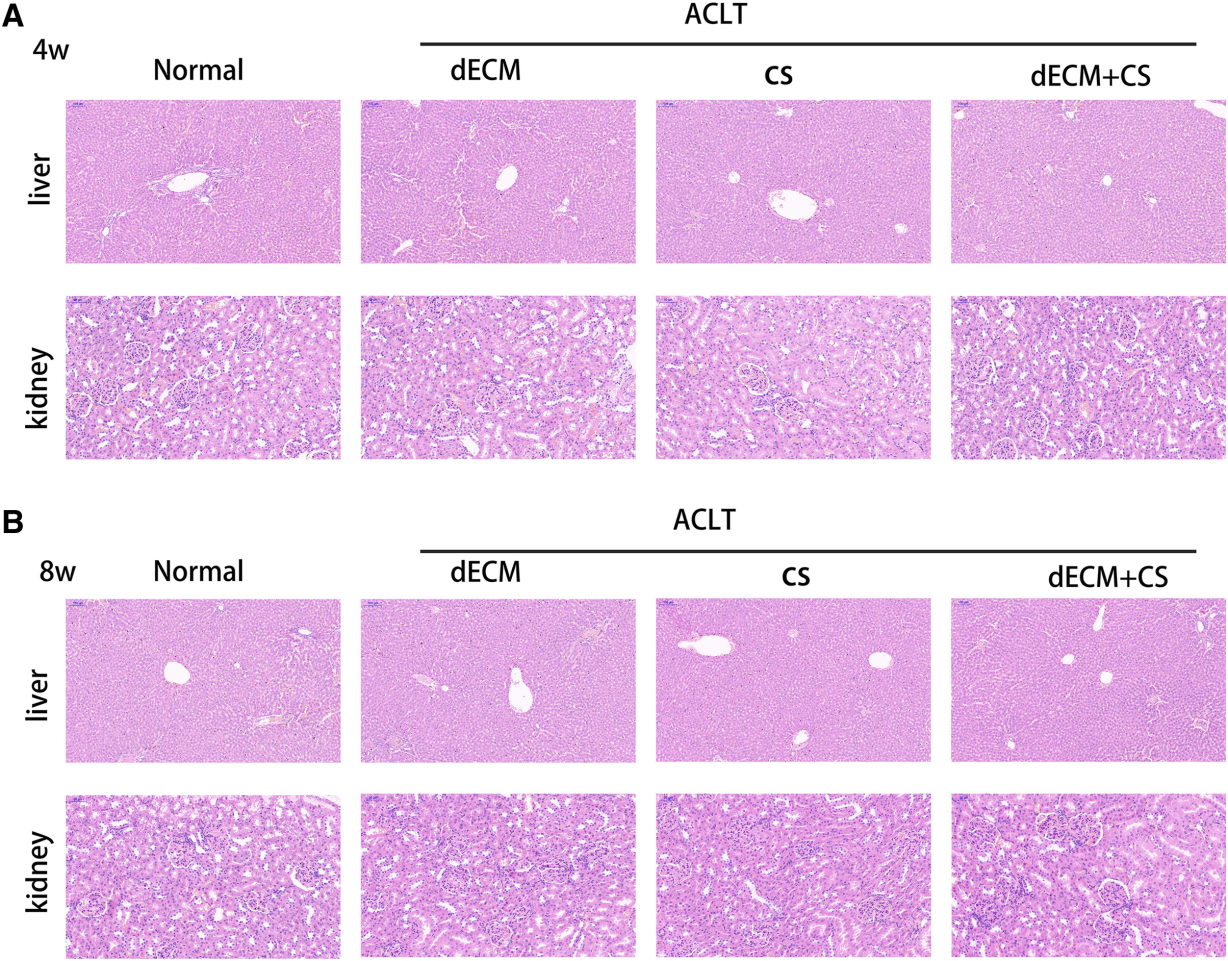
Evaluation of hepatorenal toxicity of drugs for intra-articular injection. (A and B) Liver and kidney tissue from rats in normal, dECM suspension, CS, and dECM-CS compound groups at 4 w and 8 w.

The HE staining of rat liver and kidney cells at 4 w and 8 w after the drug intervention indicated that the glomerular structure of hepatic lobules was intact, which demonstrated that no hepatorenal toxicity was produced (Fig. 7).

## Discussion

KOA refers to a common disease in orthopedics, which mostly occurs in the elderly and results in a heavy medical burden (Hunter, Schofield & Callander, 2014). At present, no drug is capable of significantly delaying or even reversing the progression of KOA. In this study, the decellularized technique was successfully applied to the rat knee cartilage, and the dECM was suggested to exert a positive effect on the growth of rat chondrocytes *in vitro*. The dECM-CS compound significantly helped relieve the pain of KOA in rats, and delayded the progression of KOA in rats. In addition, the local application of dECM-CS compound was also confirmed to have no obvious hepatorenal toxicity in rats. The results here confirmed the good effect of dECM-CS compound on the treatment of rat knee OA.

At present, the step-by-step treatment has been adopted for KOA (Abramoff & Caldera, 2020). Under more insights into the pathogenesis of KOA (Hunter & Bierma-Zeinstra, 2019) and the emergence of novel technologies (*e.g*., regenerative medicine and tissue engineering), various studies aiming at delaying the progression of KOA have emerged (Wang et al., 2017; Zhang et al., 2017). The metabolic imbalance of cartilage tissue is a vital link in the pathogenesis of OA (Rahmati et al., 2017). When chondrocytes are injured by trauma, inflammation or other factors, the synthesis and metabolism of chondrocytes are destroyed, the ECM components of cartilage are reduced, and the homeostasis of cartilage tissue is unbalanced, which causes the cartilage destruction to be gradually aggravated. Existing studies confirmed that the interaction between ECM and chondrocytes affects the differentiation and growth of chondrocytes (Gentili & Cancedda, 2009). The decrease of transcriptional level of Col2A1, ACAN, PRG4 and Sox9 are closely related to OA (Nham et al., 2019; Ikegawa et al., 2000; Haag, Gebhard & Aigner, 2008). The dECM was used in this study to interfere with rat chondrocytes, and the expressions of the mentioned genes were suggested to be up-regulated. This result indirectly indicated the protective effect of dECM on chondrocytes.

Decellularized technology acts as the premise of the application of ECM. It is capable of reducing the immunogenicity of the tissue, while retaining the composition, structure and function of the ECM (Cramer & Badylak, 2020). At present, the bone and cartilage decellularized technology has been applied in the tissue engineering technology, and it has achieved good research results (Kheir et al., 2011). In this study, the improved method was adopted to remove cells from cartilage tissue to obtain dECM (Zhang et al., 2020). This method was continuously used to treat rat knee cartilage. The rat knee cartilage was evaluated by the HE staining, the alcian blue staining and the scanning electron microscope, and the content of GAG and DNA in rat knee cartilage tissue were quantitatively detected before and after the acellular by using the biochemical methods. Next, a satisfactory dECM of rat knee cartilage was successfully obtained. To determine the effect of allogeneic cartilage dECM on the viability of chondrocytes, the *in vitro* experiments were performed, in which rat cartilage dECM was co-cultured with rat chondrocytes to observe the viability of chondrocytes. It was reported that chondrocytes grew well on cartilage dECM. Moreover, alamar blue was used to detect the toxicity of dECM to chondrocytes. As reported from the results, dECM exhibited no cytotoxicity to chondrocytes, indicating that allogeneic cartilage dECM did not adversely affect chondrocyte viability, and it was feasible to be used as a tissue engineering material for treating rat KOA.

In our previous study, we found that rabbit knee cartilage dECM can significantly delay the progression of KOA (Zhang et al., 2020). This study hopes to combine cartilage dECM with other tissue engineering materials in the study of KOA. CS is characterized by non-toxic, anti-inflammatory, good biocompatibility, slow degradation rate, etc. (Comblain et al., 2017). As a tissue engineering material, it has been maturely applied in the basic research of orthopaedics. An experimental study termed as Chitosan-cartilage extracellular matrix hybrid scaffold induces chondrogenic differentiation to adipose-derived stem cells showed that adipose stem cells differentiated into chondrocytes in CS-dECM composite three-dimensional scaffolds and built considerable cartilage extracellular matrixes (Lin et al., 2020). Thus far, there has been no report on the efficacy of CS and dECM for treating KOA. As reported from the HE staining, the alcian blue staining and the saffron fast green staining, dECM-CS compound could reduce the degree of cartilage surface fibrosis, protect chondrocytes, avoid the degradation of the extracellular matrix in the cartilage tissue, and significantly delay the progression of KOA in the rats. The OARSI pathological grade score of dECM-CS group also objectively reflected its protective effect on cartilage.

Some of the most important growth factors like TGFb, FGF, and IGF is retained in dECM. And the cartilage tissue is naturally inclined to respond to the growth factors (Vinatier et al., 2009). Cartilage tissue naturally lacks a supply of appropriate growth factors and nutrients owing to its avascular nature, so the retention of bioactive molecules will be especially beneficial in regenerating cartilage (Benders et al., 2013). These bioactive molecules may be involved in the up-regulation of genes related to cartilage synthesis and metabolism.

Type II collagen and GAGs play an important role in cartilage formation *in vivo* and *in vitro*. Biomaterials containing chitosan can provide a suitable biochemical and biomechanical environment for chondrocytes to produce type II collagen and GAGs (Muzzarelli et al., 2012). From the above, it can be inferred that dECM-CS complex plays a synergistic role in the repair of cartilage tissue.

Increasing pain in knee joint can seriously limit the normal function of the knee joint, the clinical use of non-steroidal anti-inflammatory drugs still have adverse consequences on the body (Pelletier et al., 2016). The causes of knee joint pain include knee arthritis, destruction of cartilage, etc. The hot plate test and the weight-bearing asymmetry test were performed to objectively assess the knee joint pain in rat KOA model. According to this experiment, the pain in dECM-CS group in ACLT was significantly less than that in Vehicle group, which demonstrated that dECM-CS compound could be effective in relieving pain in rat KOA model.

The drug safety has always been an important aspect of concern (Cao et al., 2020). The HE staining was performed on tissue sections of liver and kidney of rats treated for 4 w and 8 w. As revealed from the results, the drugs prepared and applied here were relatively safe.

The limitation is that the details of the interaction between dECM-CS compound and KOA require further research, and we will continue to explore the biological properties of dECM-CS compound in depth.

## Conclusion

As a novel type of composite material, dECM-CS compound has achieved good results for treating KOA in rats. it is a potential treatment of KOA and provides a new idea to treat KOA.

### Statistical methods

All the data in this experiment were processed and analyzed by SPSS19.0. Pairwise comparisons were performed by *t*-test, and comparisons between multiple groups were performed by one-way ANOVA. The data were expressed as mean ± standard deviation, *P* < 0.05 shows that the results are statistically different.

## Supplemental Information

10.7717/peerj.12188/supp-1 Supplemental Information 1 Author checklist.

10.7717/peerj.12188/supp-2 Supplemental Information 2 Raw data.

## Additional Information and Declarations

### Data Availability

The following information was supplied regarding data availability: The raw measurements are available in the Supplemental Files.

## References

[ref-1] Abramoff B, Caldera FE (2020). Osteoarthritis: pathology, diagnosis, and treatment options. Medical Clinics of North America.

[ref-2] Adali T, Kalkan R, Karimizarandi L (2019). The chondrocyte cell proliferation of a chitosan/silk fibroin/egg shell membrane hydrogels. International Journal of Biological Macromolecules.

[ref-3] Aizah N, Chong PP, Kamarul T (2019). Early alterations of subchondral bone in the rat anterior cruciate ligament transection model of osteoarthritis. Cartilage.

[ref-4] Bannuru RR, Osani MC, Vaysbrot EE, Arden NK, Bennell K, Bierma-Zeinstra SMA, Trojian T, Underwood M, McAlindon TE (2019). OARSI guidelines for the non-surgical management of knee, hip, and polyarticular osteoarthritis. Osteoarthritis Cartilage.

[ref-5] Barrot M (2012). Tests and models of nociception and pain in rodents. Neuroscience.

[ref-6] Benders KE, van Weeren PR, Badylak SF, Saris DB, Dhert WJ, Malda J (2013). Extracellular matrix scaffolds for cartilage and bone regeneration. Trends in Biotechnology.

[ref-7] Bijlsma JW, Berenbaum F, Lafeber FP (2011). Osteoarthritis: an update with relevance for clinical practice. Lancet.

[ref-8] Cao P, Li Y, Tang Y, Ding C, Hunter DJ (2020). Pharmacotherapy for knee osteoarthritis: current and emerging therapies. Expert Opinion on Pharmacotherapy.

[ref-9] Carballo CB, Nakagawa Y, Sekiya I, Rodeo SA (2017). Basic science of articular cartilage. Clinics in Sports Medicine.

[ref-10] Cohen-Solal M, Funck-Brentano T, Hay E (2013). Animal models of osteoarthritis for the understanding of the bone contribution. Bonekey Reports.

[ref-11] Comblain F, Rocasalbas G, Gauthier S, Henrotin Y (2017). Chitosan: a promising polymer for cartilage repair and viscosupplementation. Bio-Medical Materials and Engineering.

[ref-12] Cramer MC, Badylak SF (2020). Extracellular matrix-based biomaterials and their influence upon cell behavior. Annals of Biomedical Engineering.

[ref-13] Florea C, Malo MK, Rautiainen J, Mäkelä JT, Fick JM, Nieminen MT, Jurvelin JS, Davidescu A, Korhonen RK (2015). Alterations in subchondral bone plate, trabecular bone and articular cartilage properties of rabbit femoral condyles at 4 weeks after anterior cruciate ligament transection. Osteoarthritis Cartilage.

[ref-14] Gentili C, Cancedda R (2009). Cartilage and bone extracellular matrix. Current Pharmaceutical Design.

[ref-15] Haag J, Gebhard PM, Aigner T (2008). SOX gene expression in human osteoarthritic cartilage. Pathobiology.

[ref-16] Hamilton CB, Pest MA, Pitelka V, Ratneswaran A, Beier F, Chesworth BM (2015). Weight-bearing asymmetry and vertical activity differences in a rat model of post-traumatic knee osteoarthritis. Osteoarthritis Cartilage.

[ref-17] Hunter DJ, Bierma-Zeinstra S (2019). Osteoarthritis. Lancet.

[ref-18] Hunter DJ, Schofield D, Callander E (2014). The individual and socioeconomic impact of osteoarthritis. Nature Reviews Rheumatology.

[ref-19] Ikegawa S, Sano M, Koshizuka Y, Nakamura Y (2000). Isolation, characterization and mapping of the mouse and human PRG4 (proteoglycan 4) genes. Cytogenetics and Cell Genetics.

[ref-20] Kean T, Thanou M (2010). Thanou, Biodegradation, biodistribution and toxicity of chitosan. Advanced Drug Delivery Reviews.

[ref-21] Kheir E, Stapleton T, Shaw D, Jin Z, Fisher J, Ingham E (2011). Development and characterization of an acellular porcine cartilage bone matrix for use in tissue engineering. Journal of Biomedical Materials Research Part A.

[ref-22] Lee J, Hong J, Kim W, Kim GH (2020). Bone-derived dECM/alginate bioink for fabricating a 3D cell-laden mesh structure for bone tissue engineering. Carbohydrate Polymers.

[ref-23] Lin IC, Wang TJ, Wu CL, Lu DH, Chen YR, Yang KC (2020). Chitosan-cartilage extracellular matrix hybrid scaffold induces chondrogenic differentiation to adipose-derived stem cells. Regenerative Therapy.

[ref-24] Mandl LA (2019). Osteoarthritis year in review 2018: clinical. Osteoarthritis Cartilage.

[ref-25] Martel-Pelletier J, Barr AJ, Cicuttini FM, Conaghan PG, Cooper C, Goldring MB, Goldring SR, Jones G, Teichtahl AJ, Pelletier JP (2016). Osteoarthritis. Nature Reviews Disease Primers.

[ref-26] Meiyappan KP, Cote MP, Bozic KJ, Halawi MJ (2020). Adherence to the American academy of orthopaedic surgeons clinical practice guidelines for nonoperative management of knee osteoarthritis. Journal of Arthroplasty.

[ref-27] Muzzarelli RA, Greco F, Busilacchi A, Sollazzo V, Gigante A (2012). Chitosan, hyaluronan and chondroitin sulfate in tissue engineering for cartilage regeneration: a review. Carbohydr Polym.

[ref-28] Nham GTH, Zhang X, Asou Y, Shinomura T (2019). Expression of type II collagen and aggrecan genes is regulated through distinct epigenetic modifications of their multiple enhancer elements. Gene.

[ref-29] Pelletier JP, Martel-Pelletier J, Rannou F, Cooper C (2016). Efficacy and safety of oral NSAIDs and analgesics in the management of osteoarthritis: evidence from real-life setting trials and surveys. Seminars in Arthritis and Rheumatism.

[ref-30] Pritzker KP, Gay S, Jimenez SA, Ostergaard K, Pelletier JP, Revell PA, Salter D, van den Berg WB (2006). Osteoarthritis cartilage histopathology: grading and staging. Osteoarthritis Cartilage.

[ref-31] Rahmati M, Nalesso G, Mobasheri A, Mozafari M (2017). Aging and osteoarthritis: central role of the extracellular matrix. Ageing Research Reviews.

[ref-32] Ravindran S, Kotecha M, Huang CC, Ye A, Pothirajan P, Yin Z, Magin R, George A (2015). Biological and MRI characterization of biomimetic ECM scaffolds for cartilage tissue regeneration. Biomaterials.

[ref-33] Vinatier C, Mrugala D, Jorgensen C, Guicheux J, Noël D (2009). Cartilage engineering: a crucial combination of cells, biomaterials and biofactors. Trends in Biotechnology.

[ref-34] Wang W, Meng Q, Li Q, Liu J, Zhou M, Jin Z, Zhao K (2020). Chitosan derivatives and their application in biomedicine. International Journal of Molecular Sciences.

[ref-35] Wang Y, Yu D, Liu Z, Zhou F, Dai J, Wu B, Zhou J, Heng BC, Zou XH, Ouyang H, Liu H (2017). Exosomes from embryonic mesenchymal stem cells alleviate osteoarthritis through balancing synthesis and degradation of cartilage extracellular matrix. Stem Cell Research & Therapy.

[ref-36] Yao Q, Zheng YW, Lan QH, Kou L, Xu HL, Zhao YZ (2019). Recent development and biomedical applications of decellularized extracellular matrix biomaterials. Materials Science and Engineering: C.

[ref-37] Zhang Y, Dai J, Yan L, Sun Y (2020). Intra-articular injection of decellularized extracellular matrices in the treatment of osteoarthritis in rabbits. PeerJ.

[ref-38] Zhang Y, Lei Z, Qi Y, Di T, Li G, Zhang W, Yan W (2017). Adipose-derived stem cell sheet encapsulated construct of micro-porous decellularized cartilage debris and hydrogel for cartilage defect repair. Medical Hypotheses.

