# Supplementary material for: The effect of cartilage decellularized extracellular matrix-chitosan compound on treating knee osteoarthritis in rats
Source: PeerJ. 2021 Oct 12;9:e12188. doi: 10.7717/peerj.12188 (PMC8519179; doi:10.7717/peerj.12188)
Supplement: Supplemental Information 2 [file peerj-09-12188-s002.zip › The original data-Deng Chen-In English/Fig. 4/RT-PCR/Table1.docx]

Table1 Primers used for RT-qPCR

| **Genes** | **Primer sequence (5’-3’)** |
| --- | --- |
| Col2A1 | S: GAGCGGAGACTACTGGATTGATC |
|  | AS: GACGTTAGCGGTGTTGGGAG |
| ACAN | S: GGTGTCACTTCCCAACTATCCAG |
|  | AS: GGCTCGGTCAAAGTCCAGTG |
| SOX-9 | S: GCTGAAGGGCTACGACTGGA |
|  | AS: TTGCCCATTCTTCACCGACT |
| PRG4 | S: ATCTCCACCACGCAGAATCACT |
|  | AS: ACGGGCTCCTGTTTGTAAGTGT |
| GAPDH | S: CACTGTGCCCATCTACGA |
|  | AS: TGATGTCACGCACGATTT |

S sense, AS antisence.
