# Supplementary figures and images for: The effect of cartilage decellularized extracellular matrix-chitosan compound on treating knee osteoarthritis in rats
Source: PeerJ. 2021 Oct 12;9:e12188. doi: 10.7717/peerj.12188 (PMC8519179; doi:10.7717/peerj.12188)

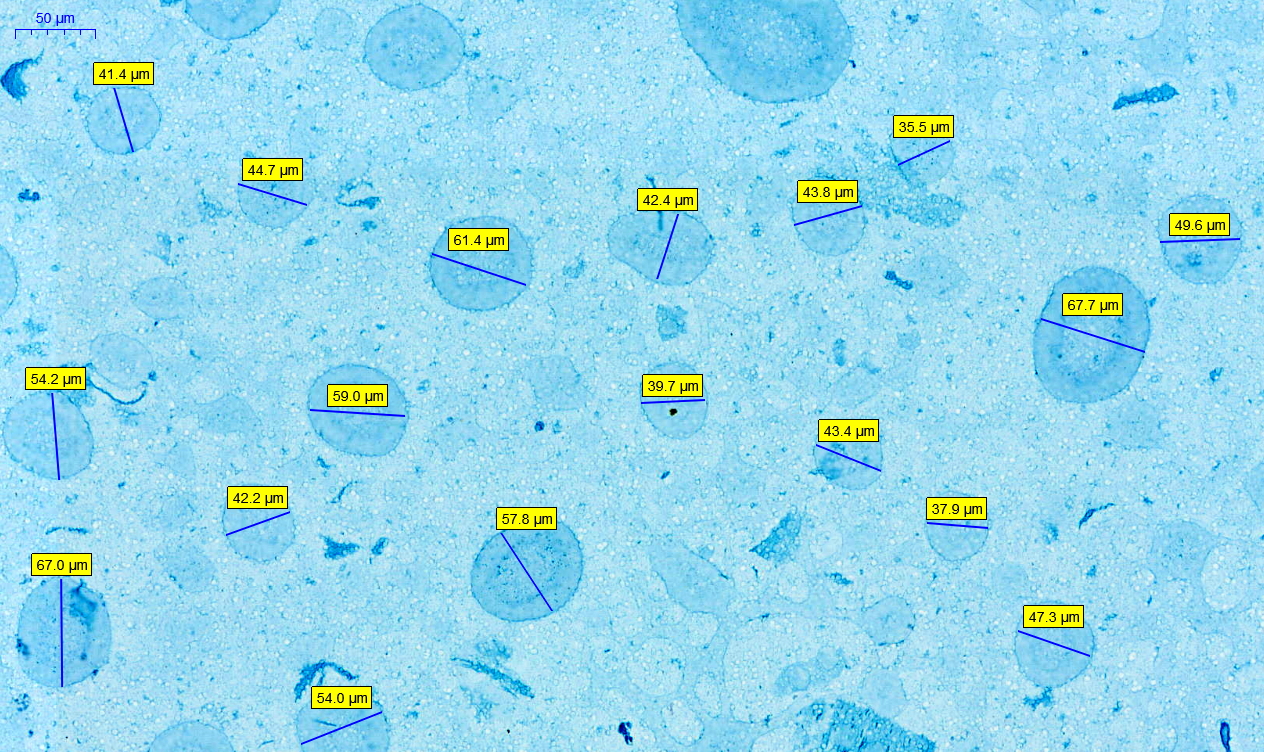

Supplement: Supplemental Information 2 [file peerj-09-12188-s002.zip › The original data-Deng Chen-In English/Fig. 2/(the dECMs particle sizes characterization by Alcian Blue).jpg]

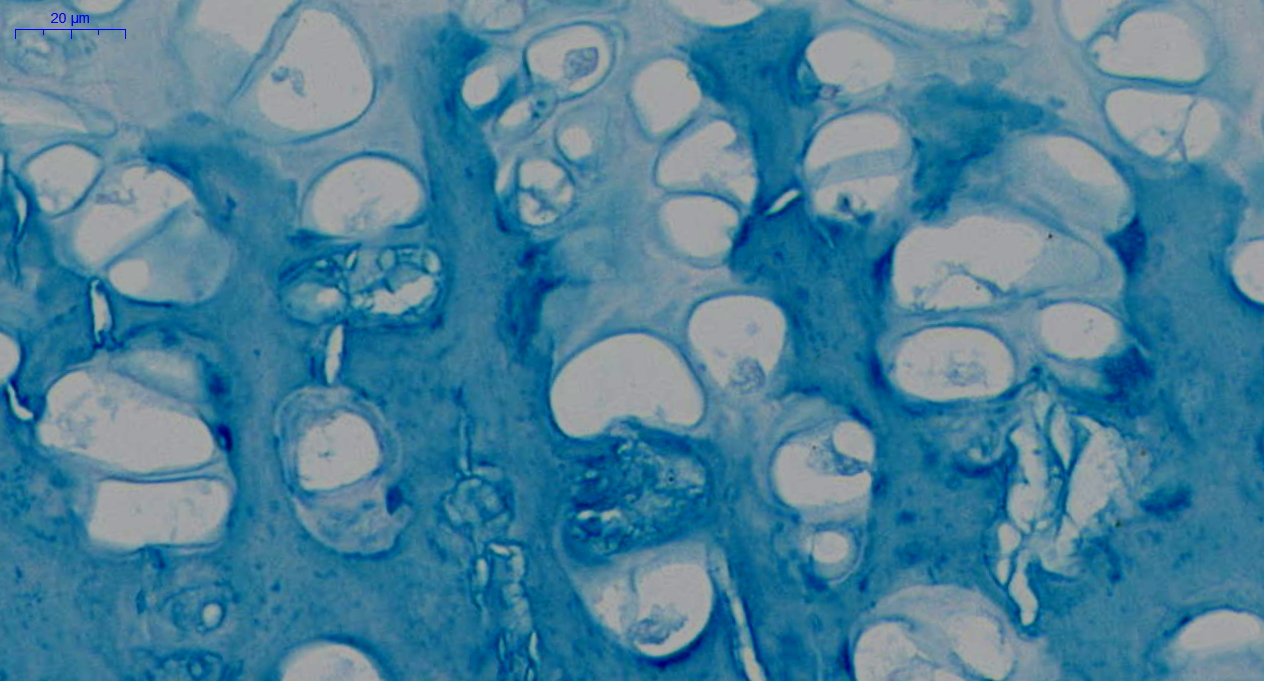

Supplement: Supplemental Information 2 [file peerj-09-12188-s002.zip › The original data-Deng Chen-In English/Fig. 2/Alcian blue after.jpg]

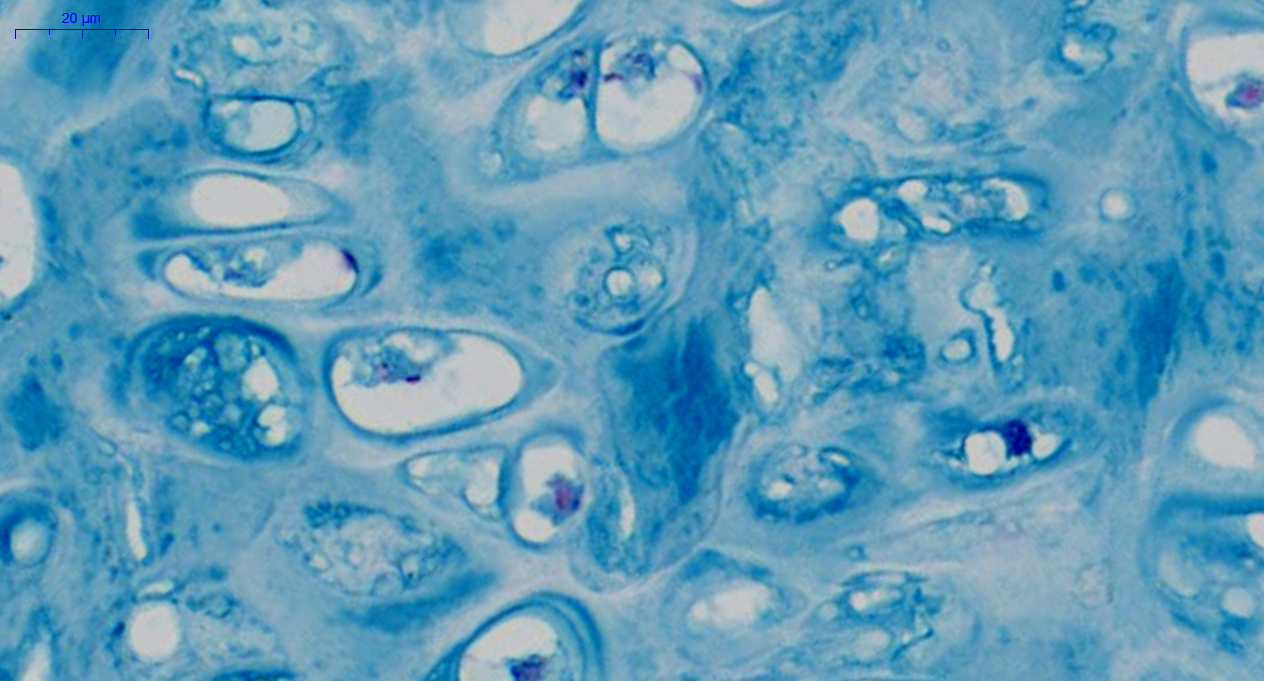

Supplement: Supplemental Information 2 [file peerj-09-12188-s002.zip › The original data-Deng Chen-In English/Fig. 2/Alcian blue before.jpg]

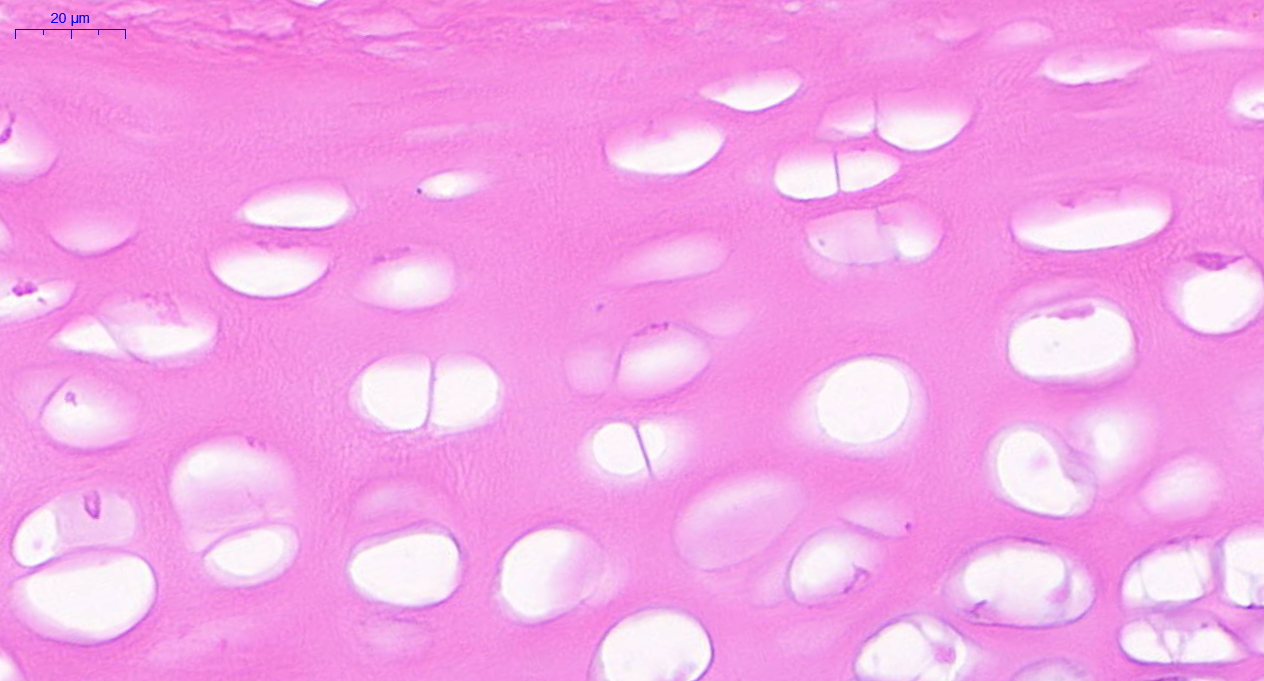

Supplement: Supplemental Information 2 [file peerj-09-12188-s002.zip › The original data-Deng Chen-In English/Fig. 2/HE after.jpg]

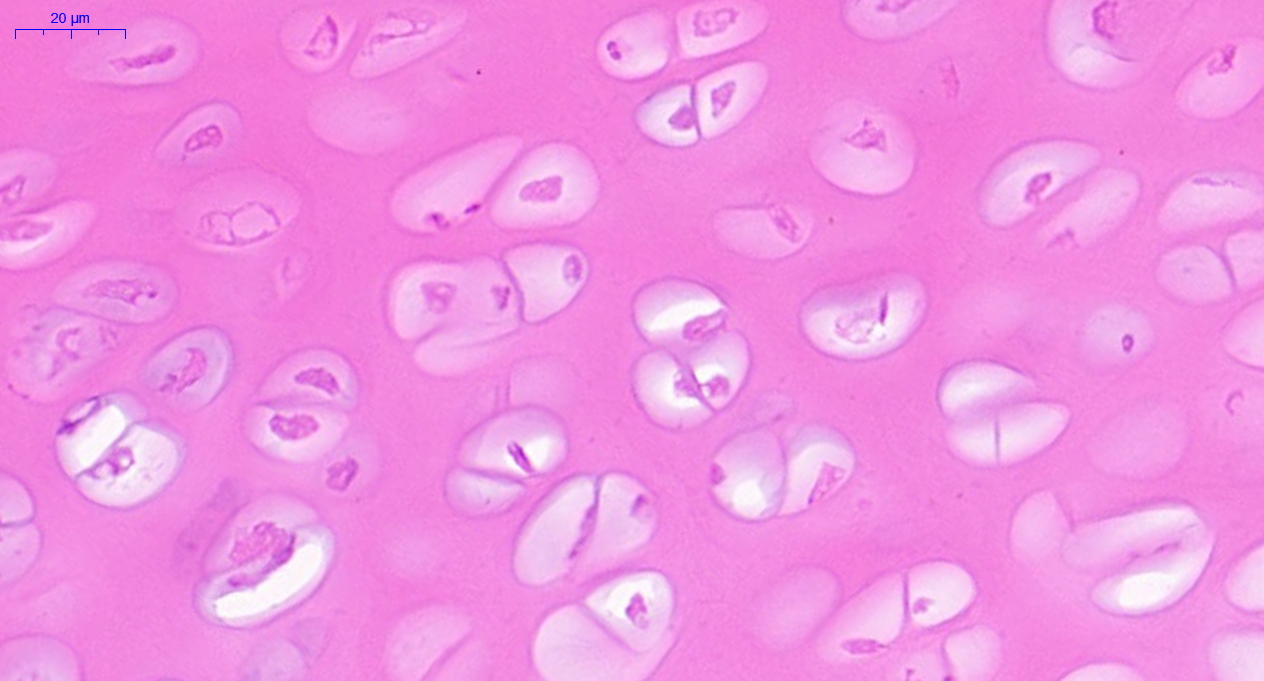

Supplement: Supplemental Information 2 [file peerj-09-12188-s002.zip › The original data-Deng Chen-In English/Fig. 2/HE before.jpg]

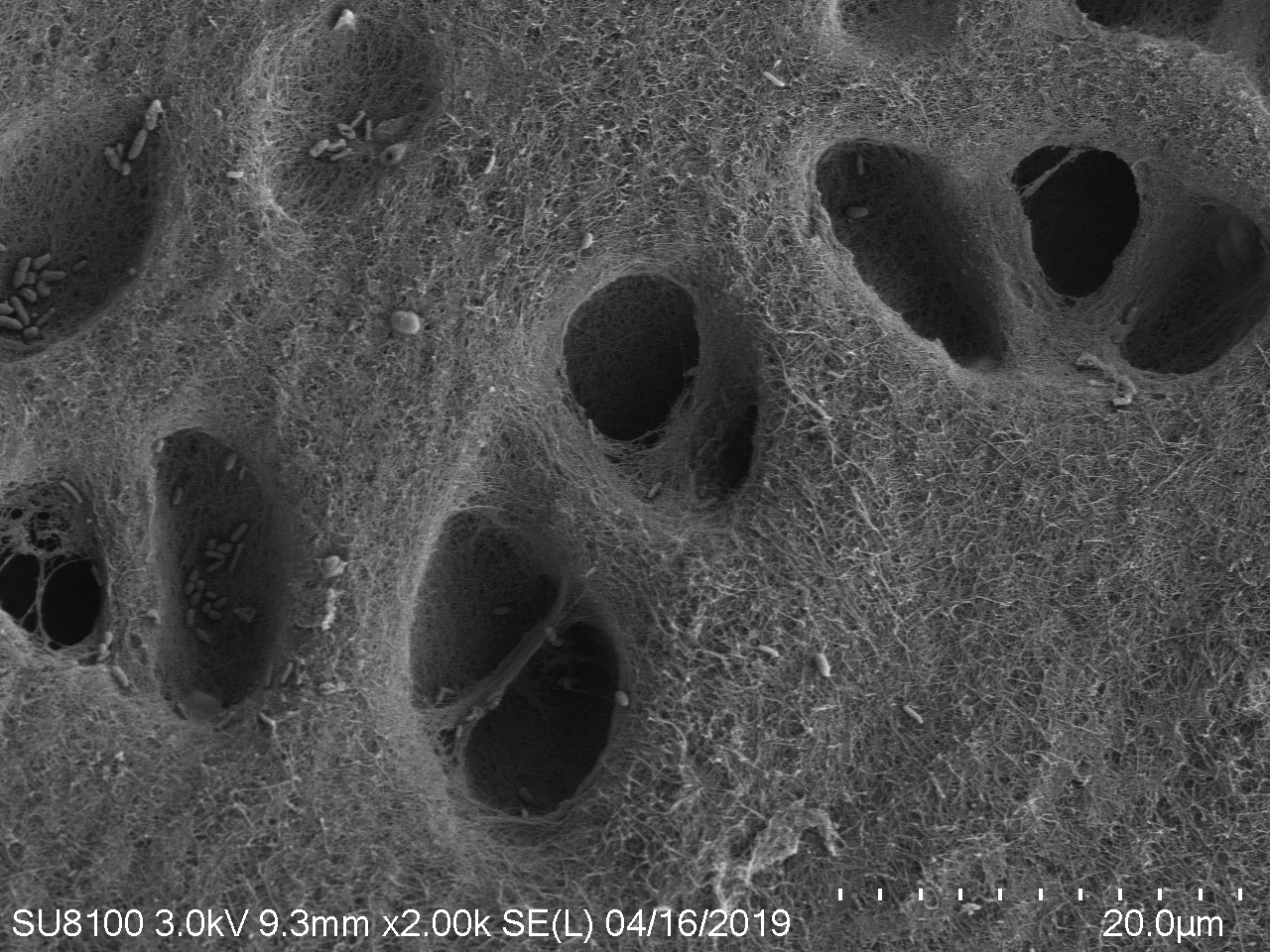

Supplement: Supplemental Information 2 [file peerj-09-12188-s002.zip › The original data-Deng Chen-In English/Fig. 2/SEM after.tif]

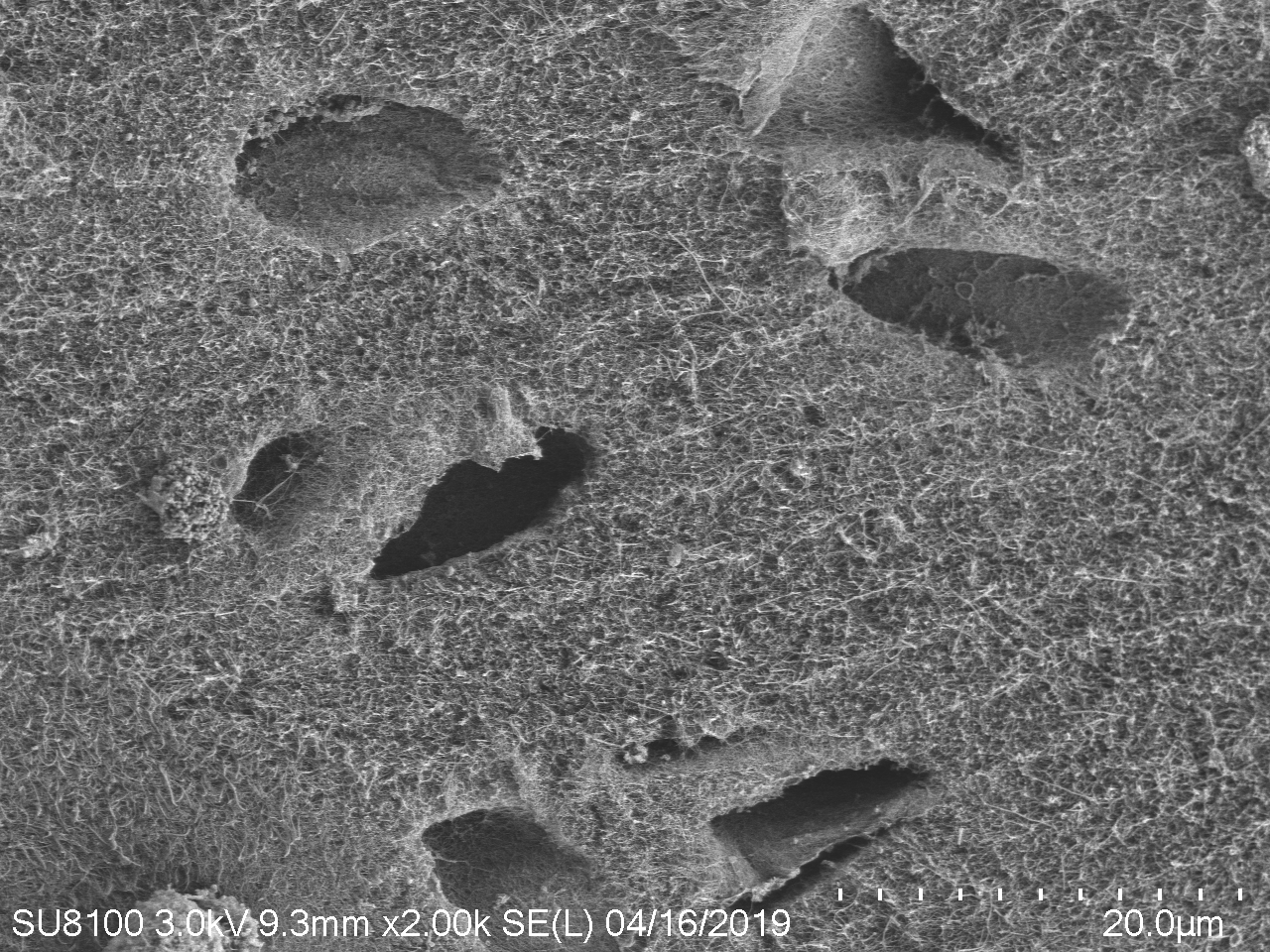

Supplement: Supplemental Information 2 [file peerj-09-12188-s002.zip › The original data-Deng Chen-In English/Fig. 2/SEM before.tif]

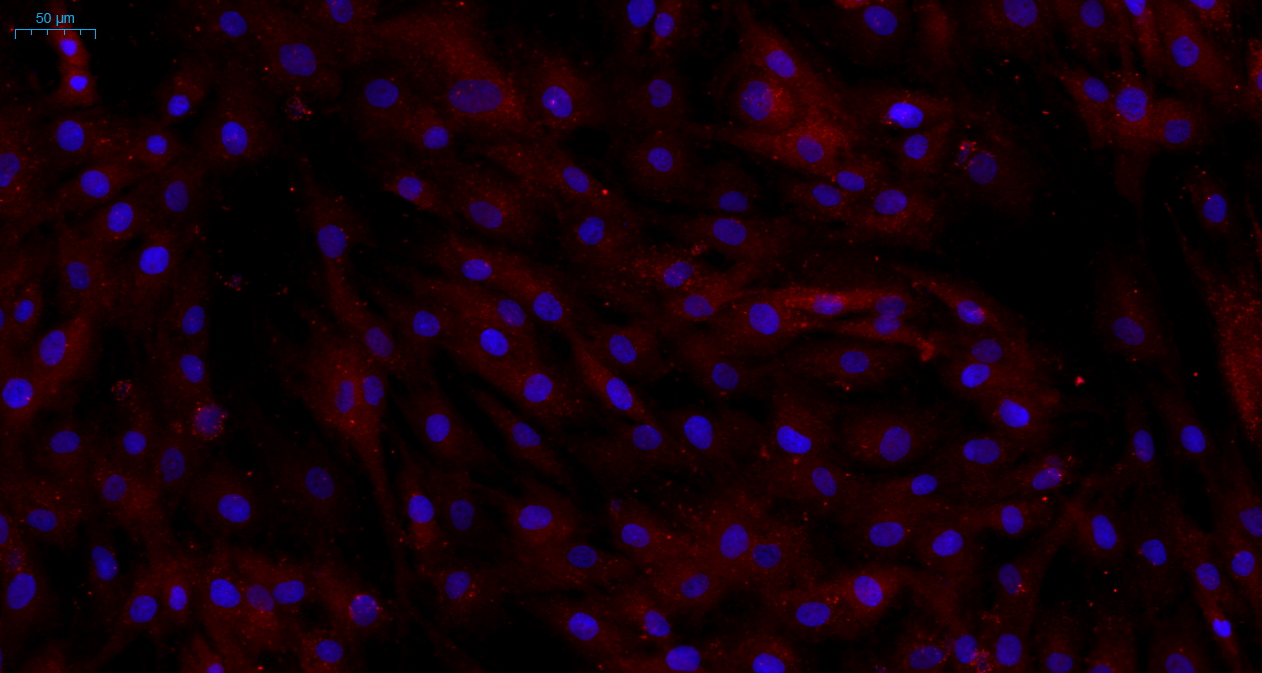

Supplement: Supplemental Information 2 [file peerj-09-12188-s002.zip › The original data-Deng Chen-In English/Fig. 3/Identification of chondrocytes/A.jpg]

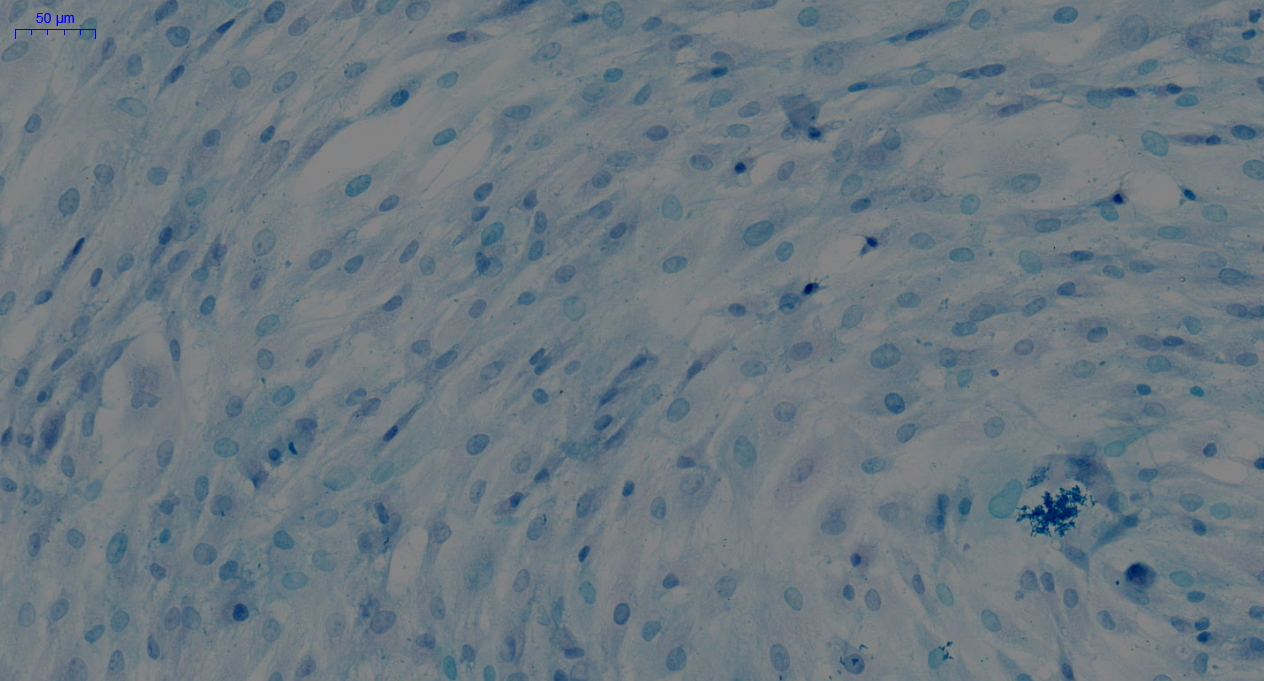

Supplement: Supplemental Information 2 [file peerj-09-12188-s002.zip › The original data-Deng Chen-In English/Fig. 3/Identification of chondrocytes/B.jpg]

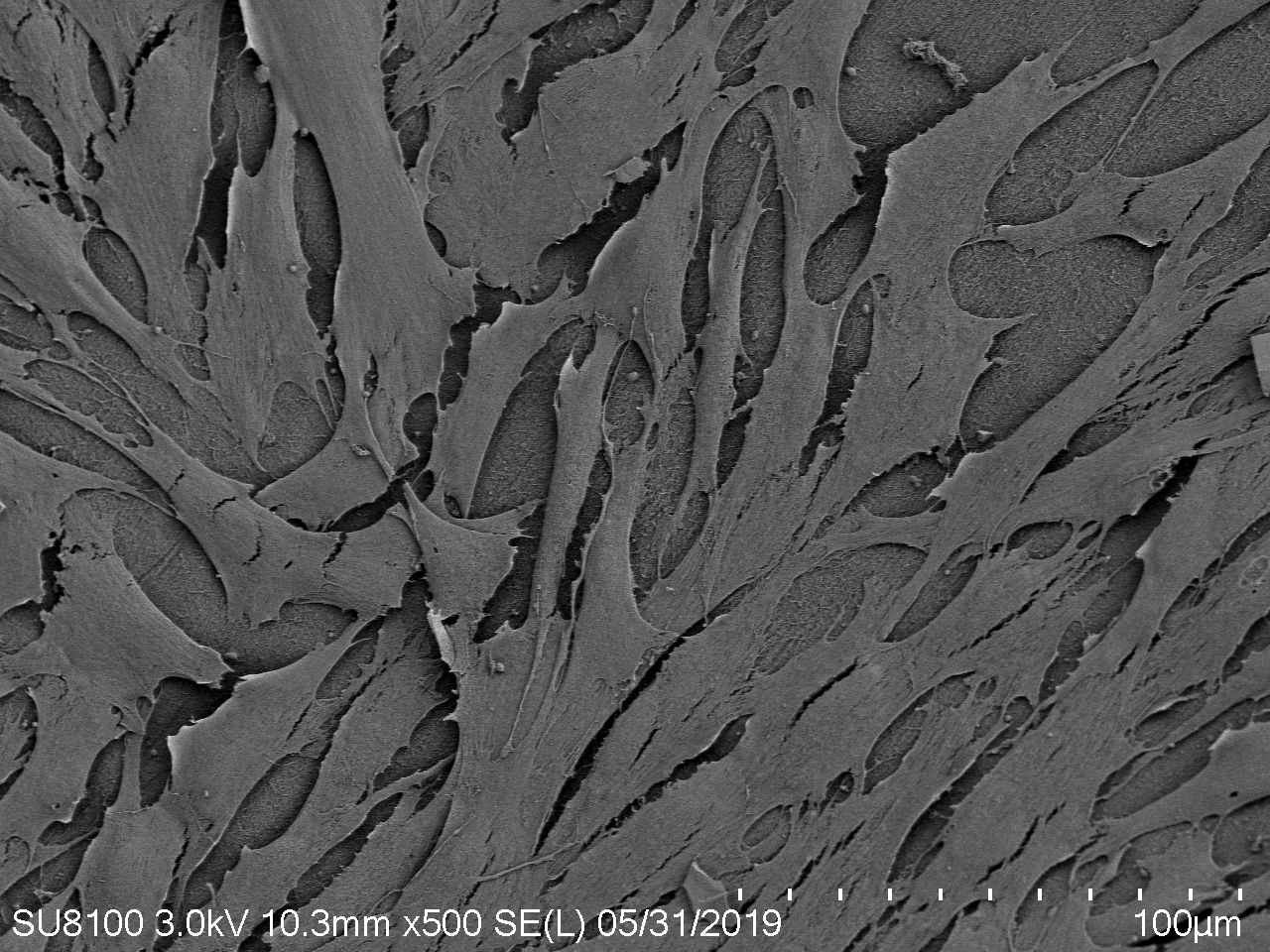

Supplement: Supplemental Information 2 [file peerj-09-12188-s002.zip › The original data-Deng Chen-In English/Fig. 3/Rat chondrocytes cultured with dECM/C.tif]

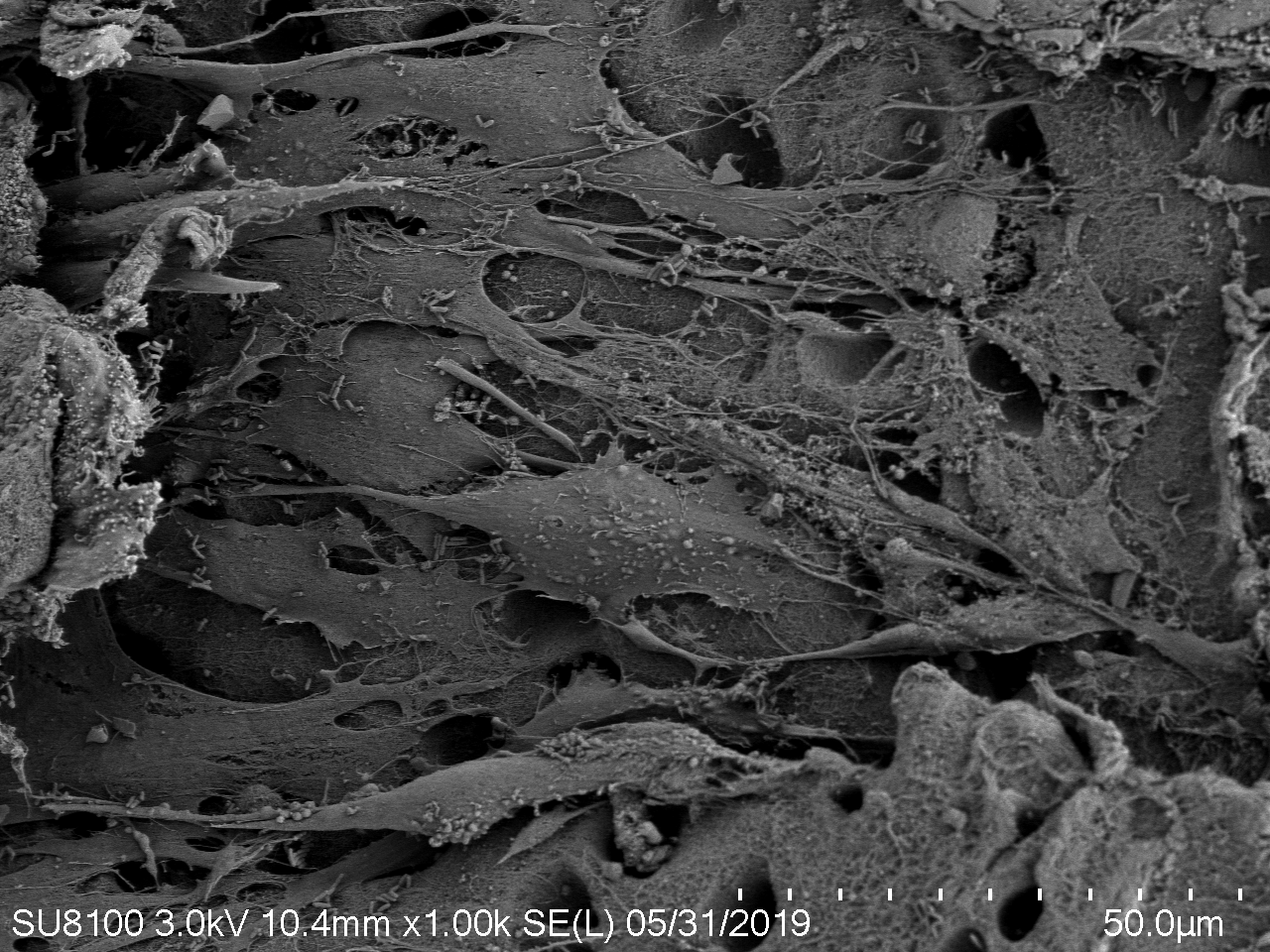

Supplement: Supplemental Information 2 [file peerj-09-12188-s002.zip › The original data-Deng Chen-In English/Fig. 3/Rat chondrocytes cultured with dECM/D.tif]

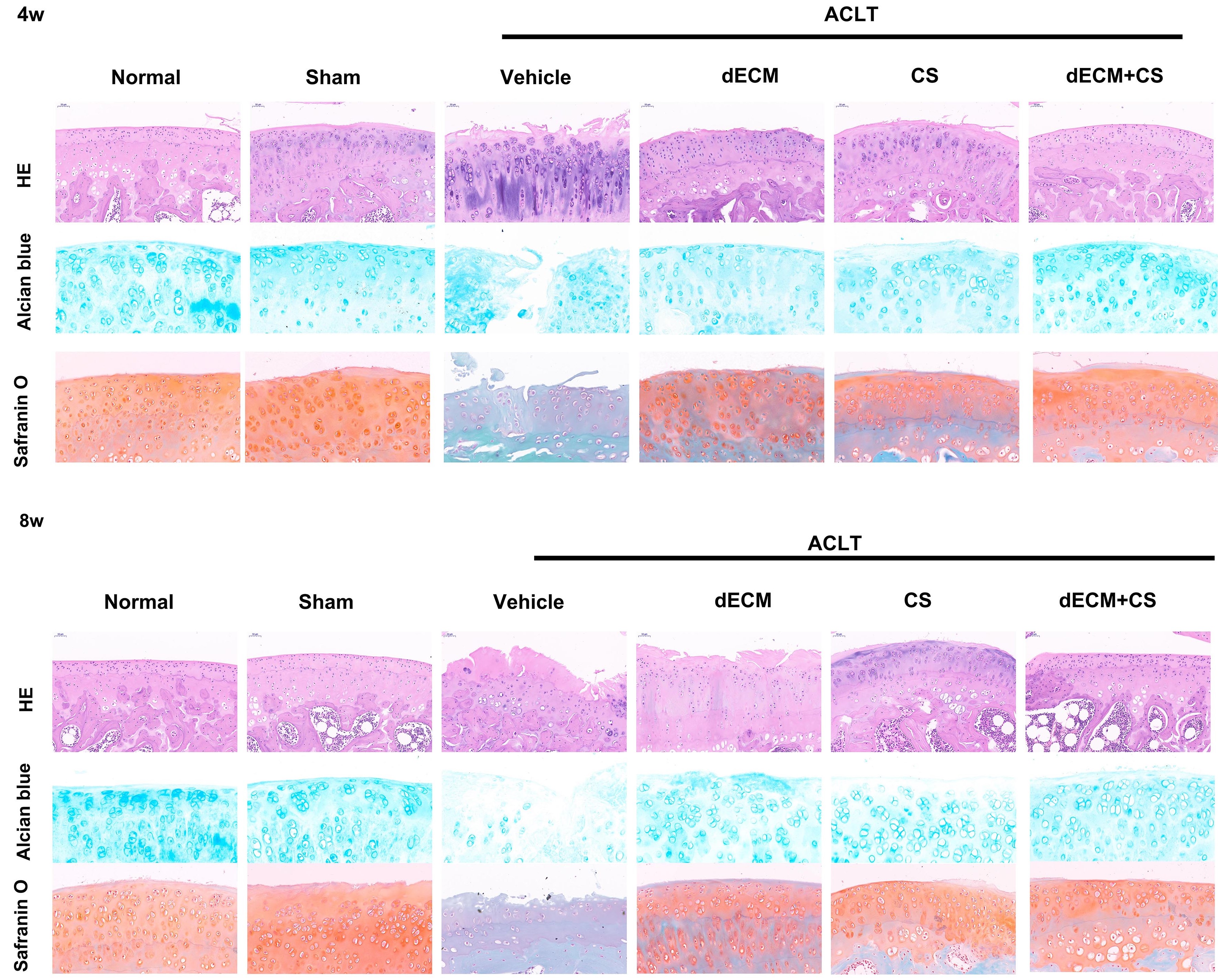

Supplement: Supplemental Information 2 [file peerj-09-12188-s002.zip › The original data-Deng Chen-In English/Fig. 5/Establishment of OA model and intra-articular injection treatment)/knee joint of rats.jpg]

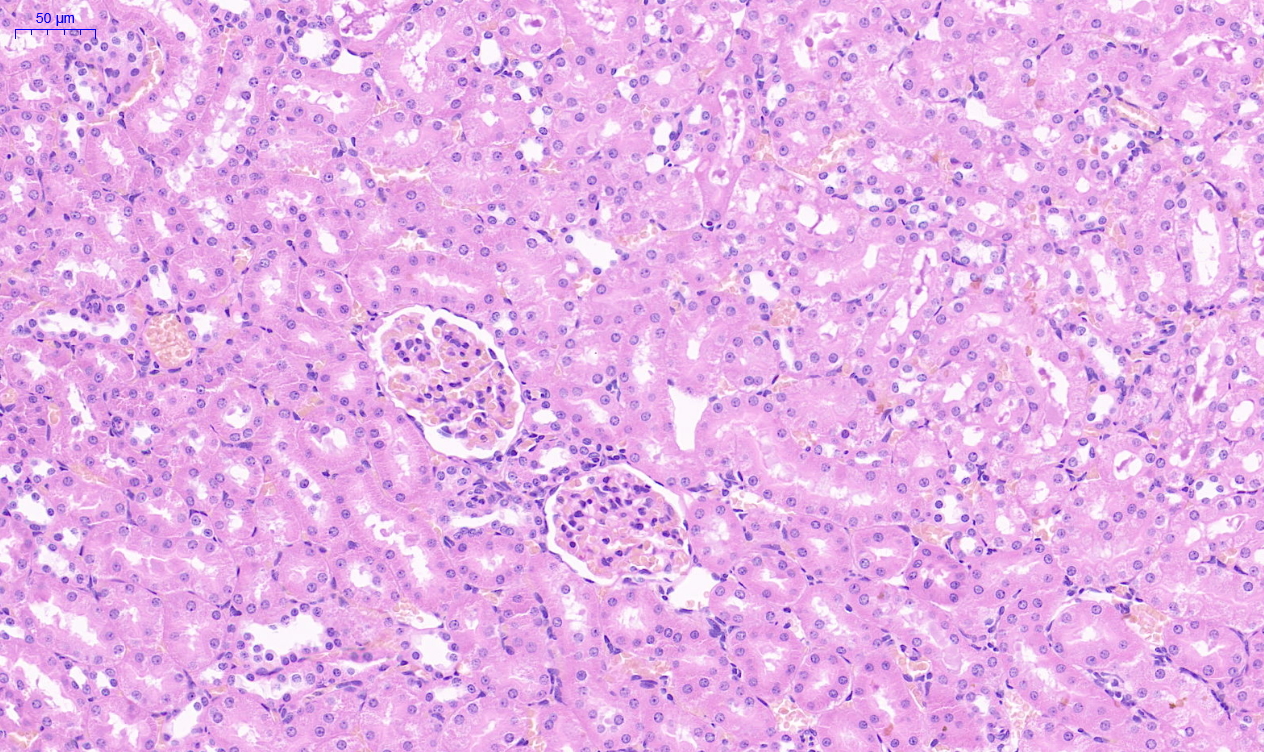

Supplement: Supplemental Information 2 [file peerj-09-12188-s002.zip › The original data-Deng Chen-In English/Fig. 7/4w kidney/CS kidney.jpg]

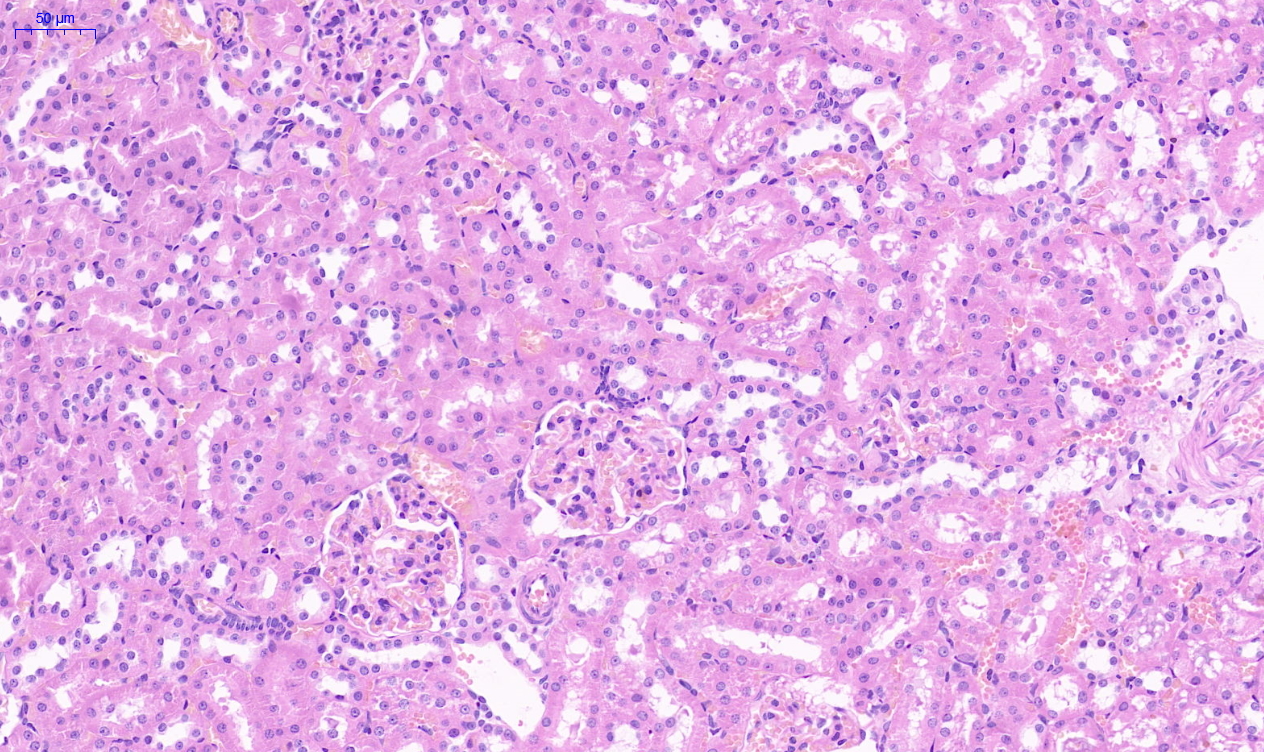

Supplement: Supplemental Information 2 [file peerj-09-12188-s002.zip › The original data-Deng Chen-In English/Fig. 7/4w kidney/dECM kidney.jpg]

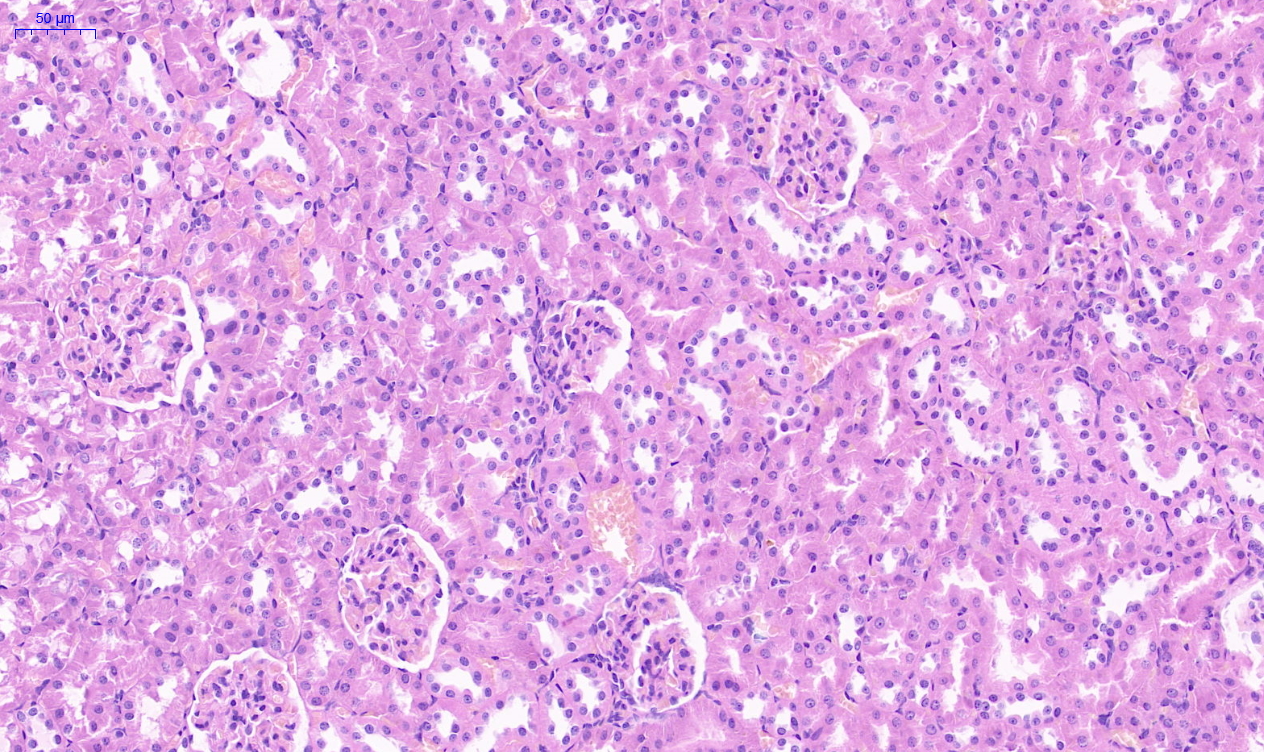

Supplement: Supplemental Information 2 [file peerj-09-12188-s002.zip › The original data-Deng Chen-In English/Fig. 7/4w kidney/dECM+CS kidney.jpg]

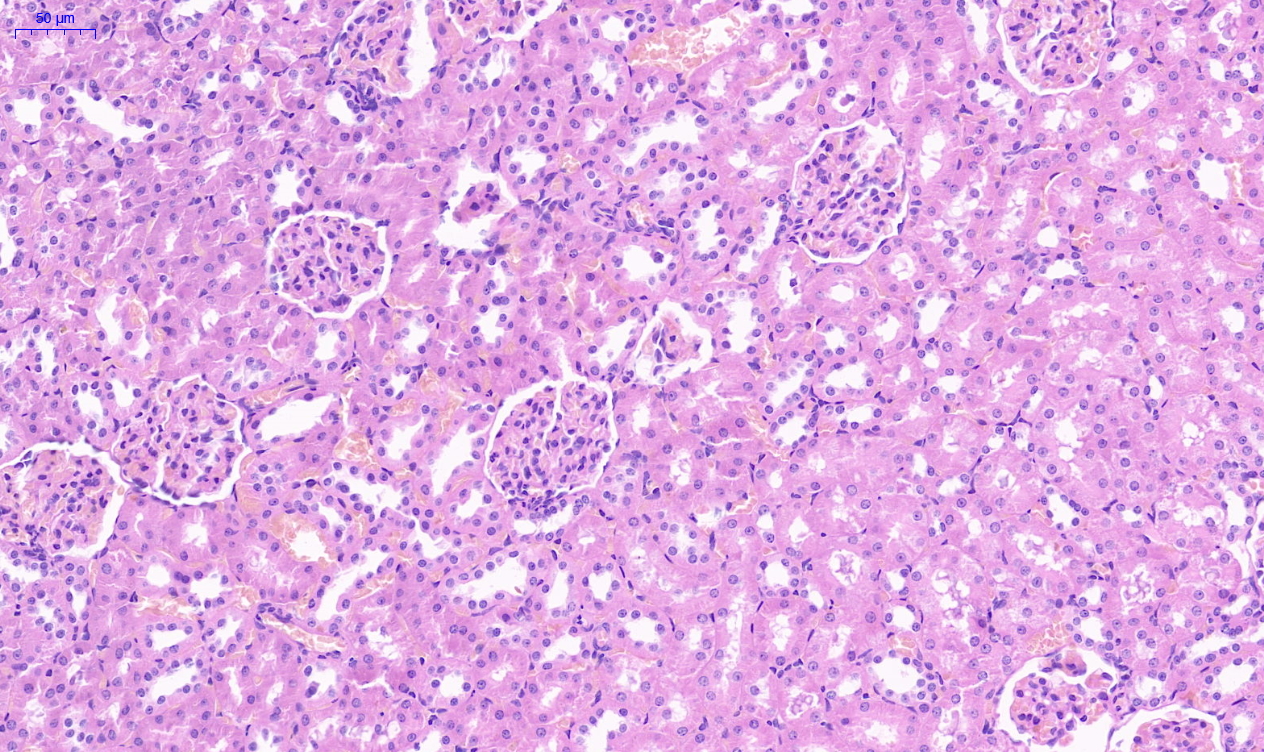

Supplement: Supplemental Information 2 [file peerj-09-12188-s002.zip › The original data-Deng Chen-In English/Fig. 7/4w kidney/normal kidney.jpg]

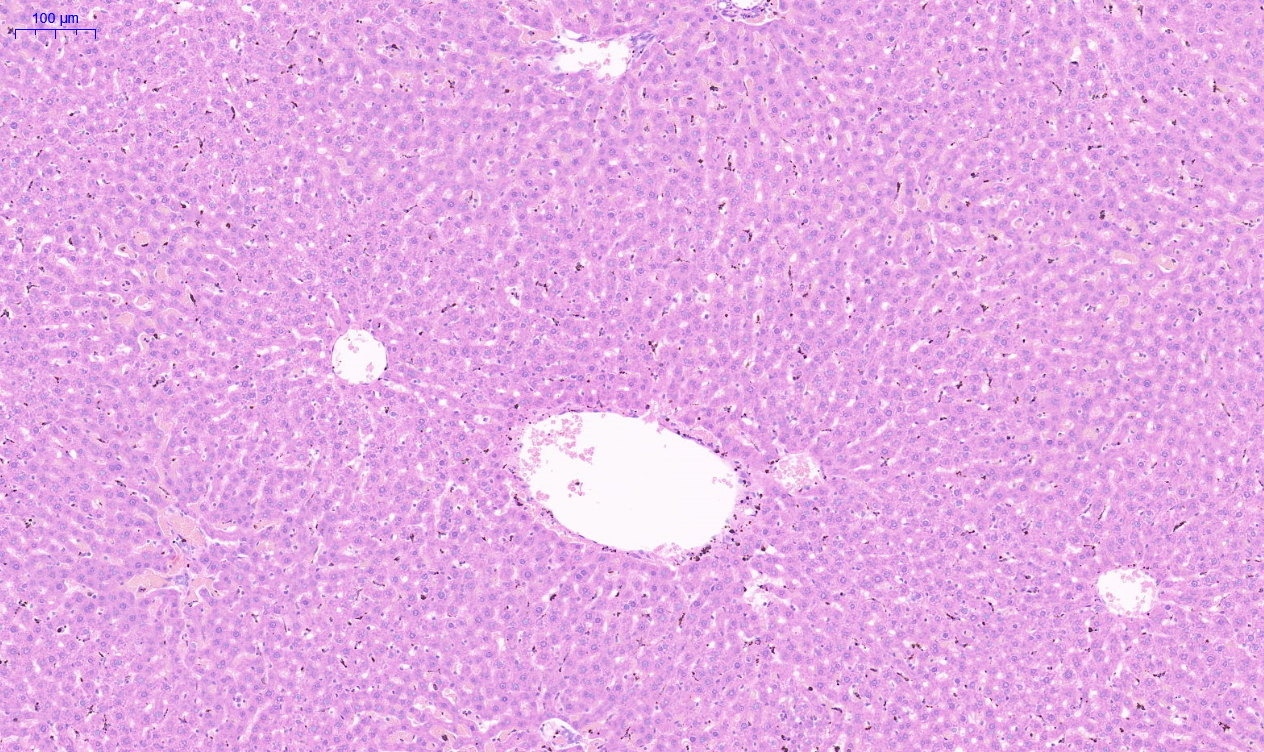

Supplement: Supplemental Information 2 [file peerj-09-12188-s002.zip › The original data-Deng Chen-In English/Fig. 7/4w liver/CS liver.jpg]

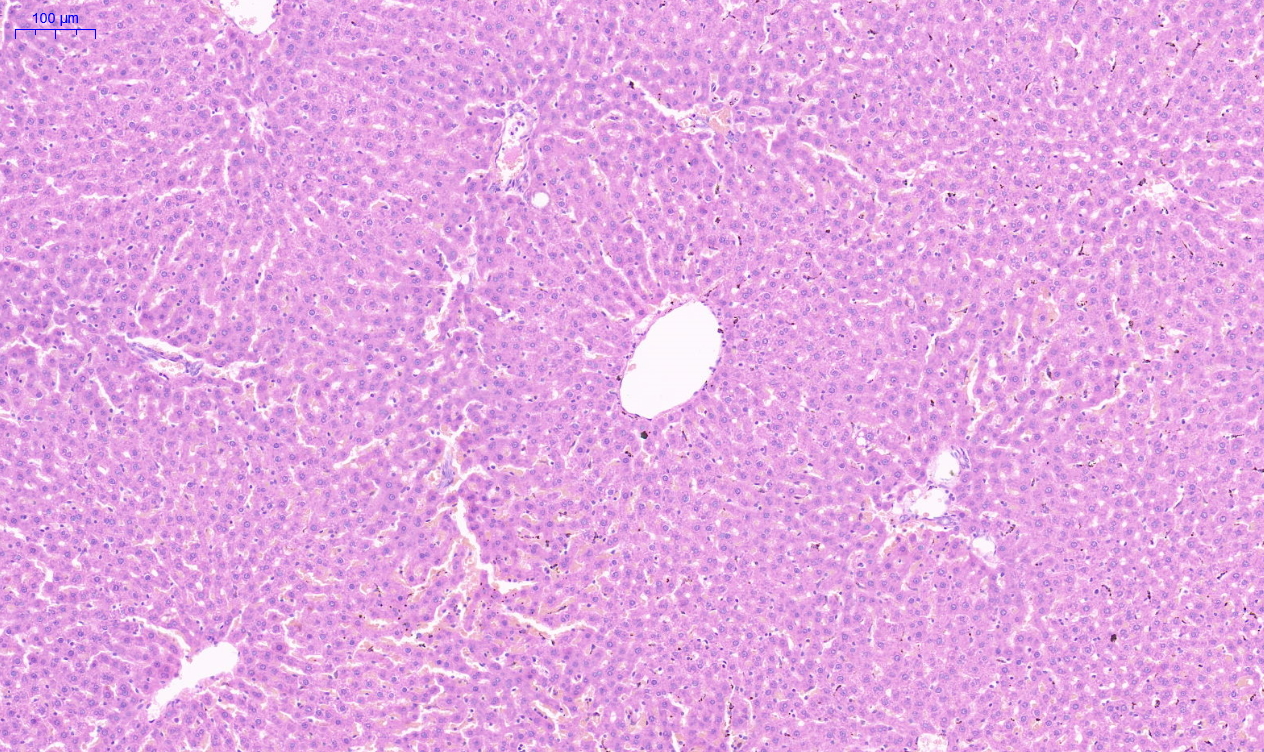

Supplement: Supplemental Information 2 [file peerj-09-12188-s002.zip › The original data-Deng Chen-In English/Fig. 7/4w liver/dECM liver.jpg]

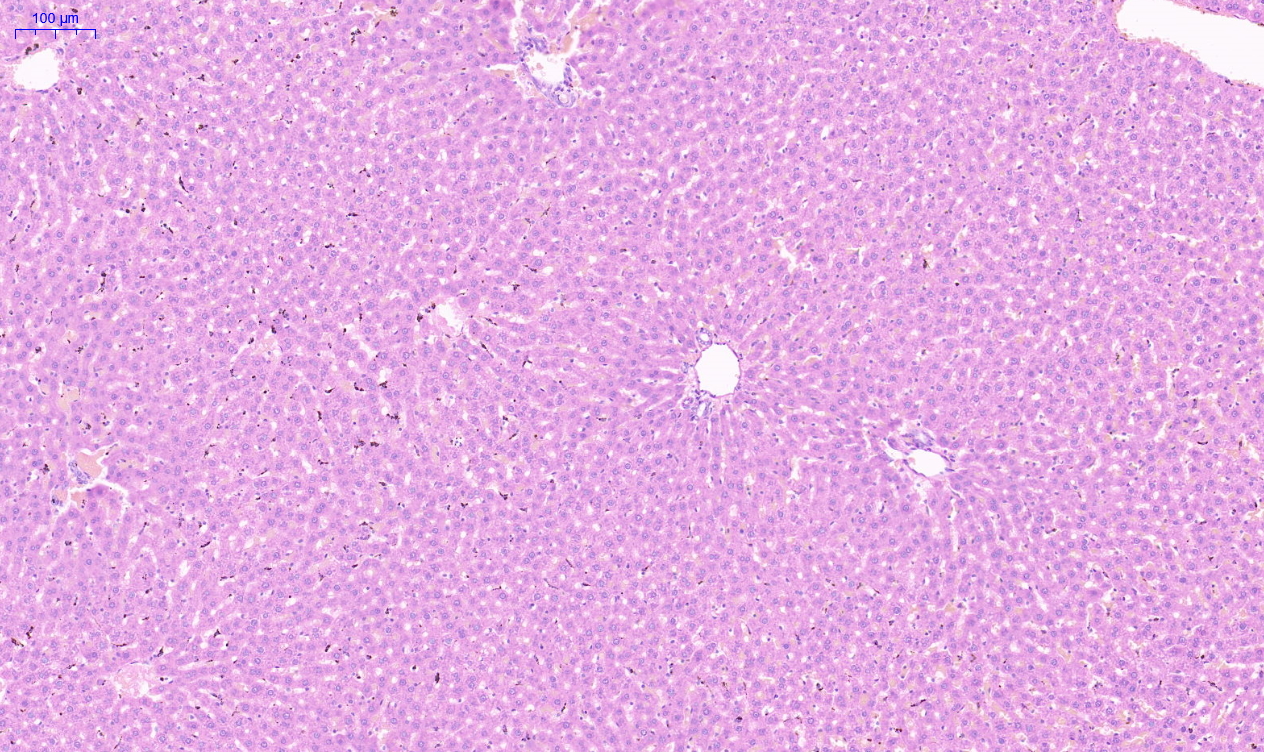

Supplement: Supplemental Information 2 [file peerj-09-12188-s002.zip › The original data-Deng Chen-In English/Fig. 7/4w liver/dECM+CS liver.jpg]

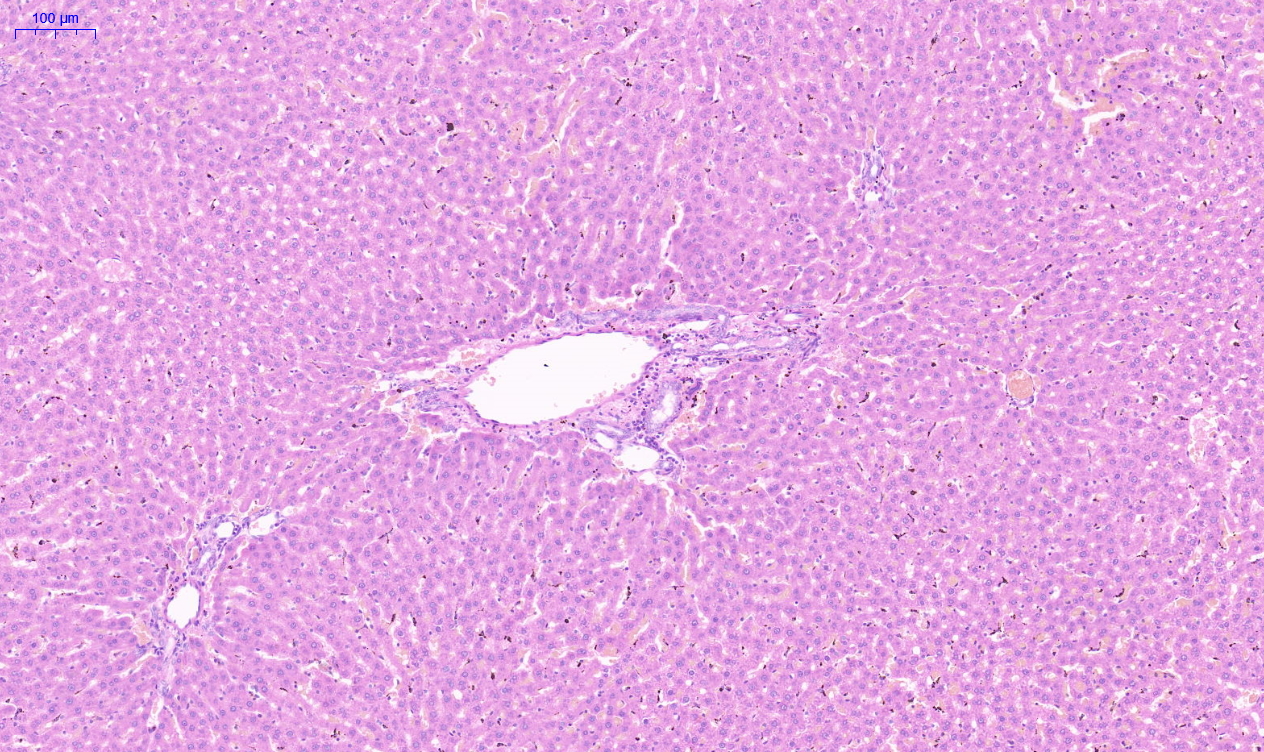

Supplement: Supplemental Information 2 [file peerj-09-12188-s002.zip › The original data-Deng Chen-In English/Fig. 7/4w liver/normal liver.jpg]

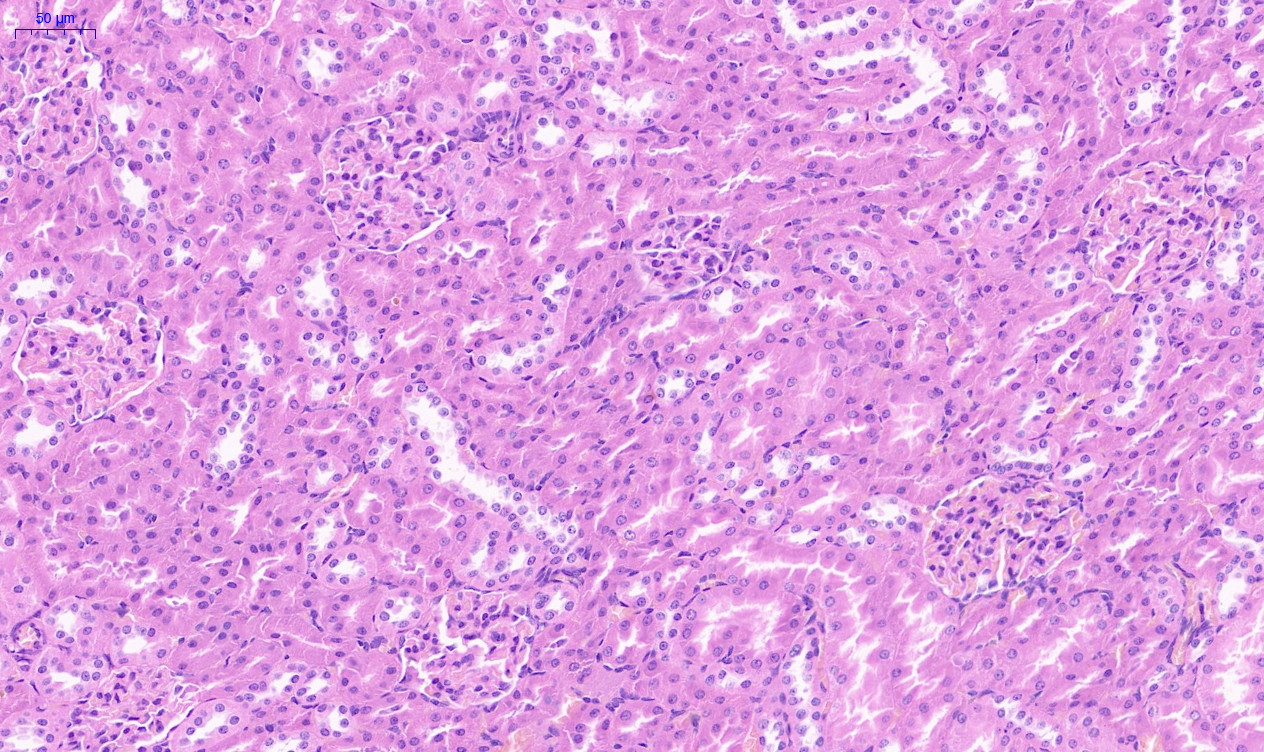

Supplement: Supplemental Information 2 [file peerj-09-12188-s002.zip › The original data-Deng Chen-In English/Fig. 7/8w kidney/CS kidney.jpg]

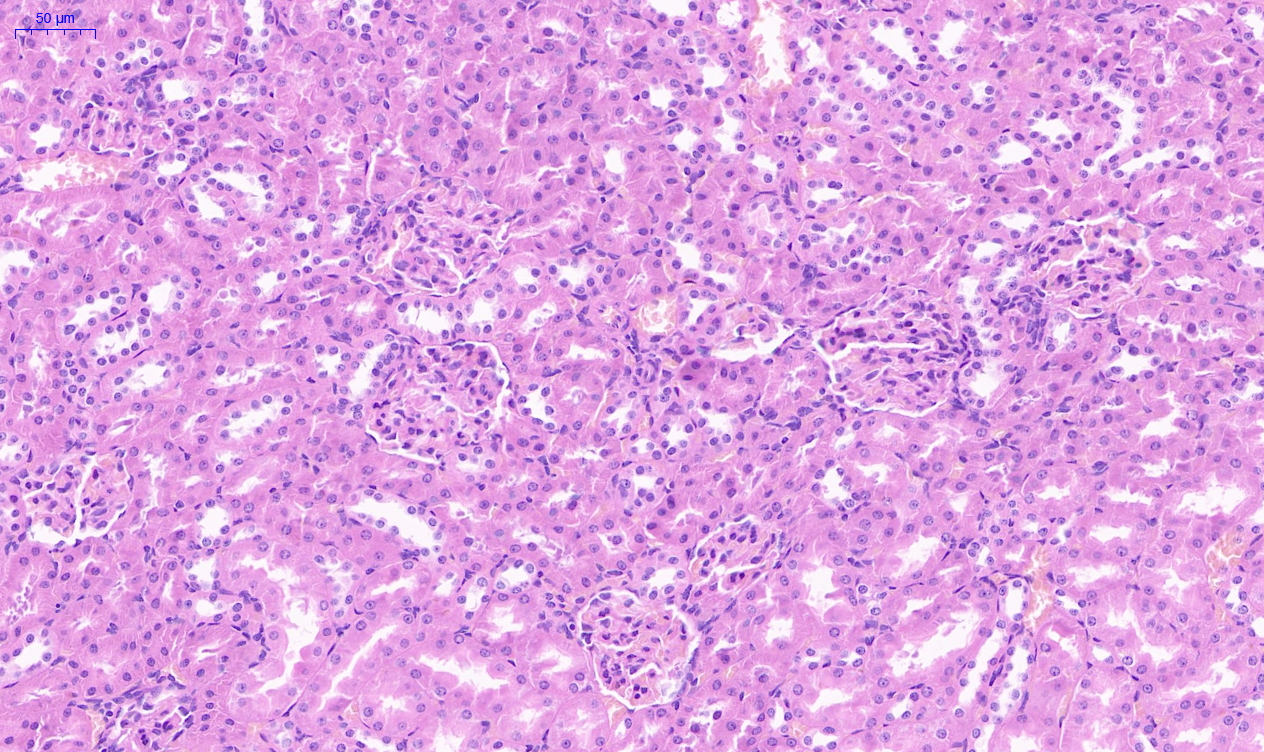

Supplement: Supplemental Information 2 [file peerj-09-12188-s002.zip › The original data-Deng Chen-In English/Fig. 7/8w kidney/dECM kidney.jpg]

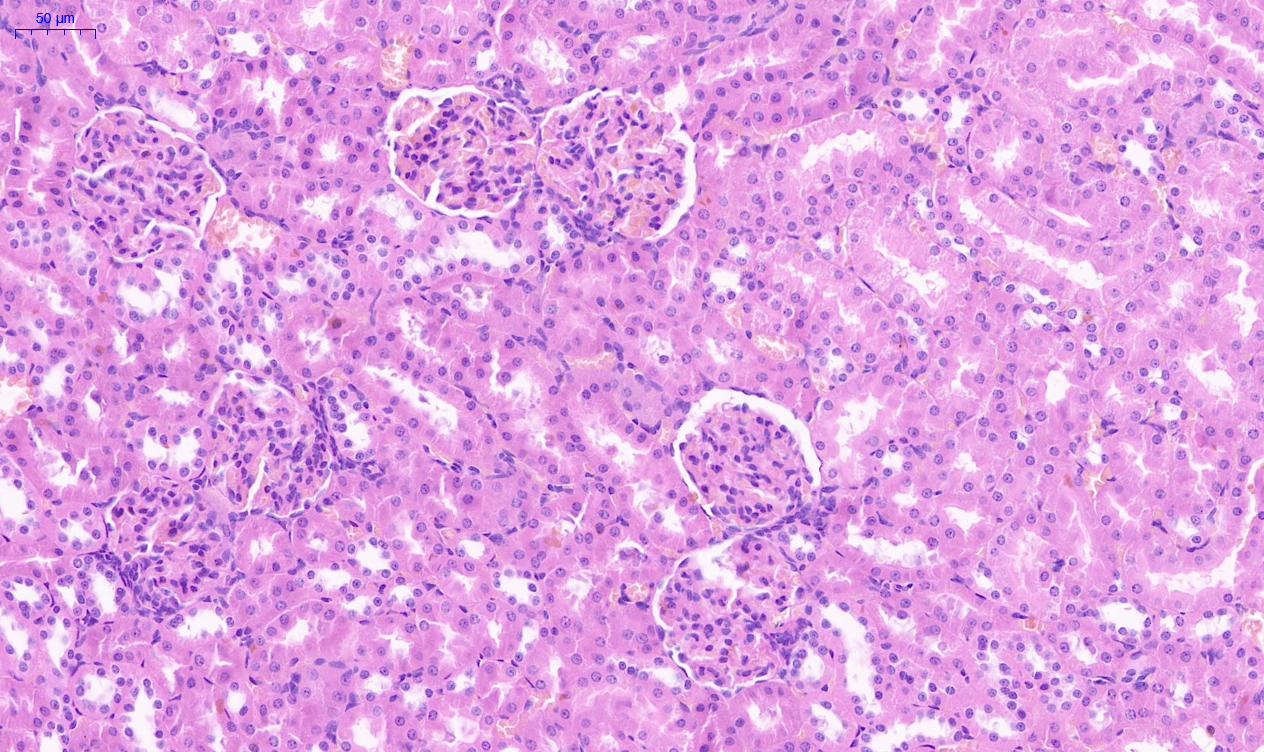

Supplement: Supplemental Information 2 [file peerj-09-12188-s002.zip › The original data-Deng Chen-In English/Fig. 7/8w kidney/dECM+CS kidney.jpg]

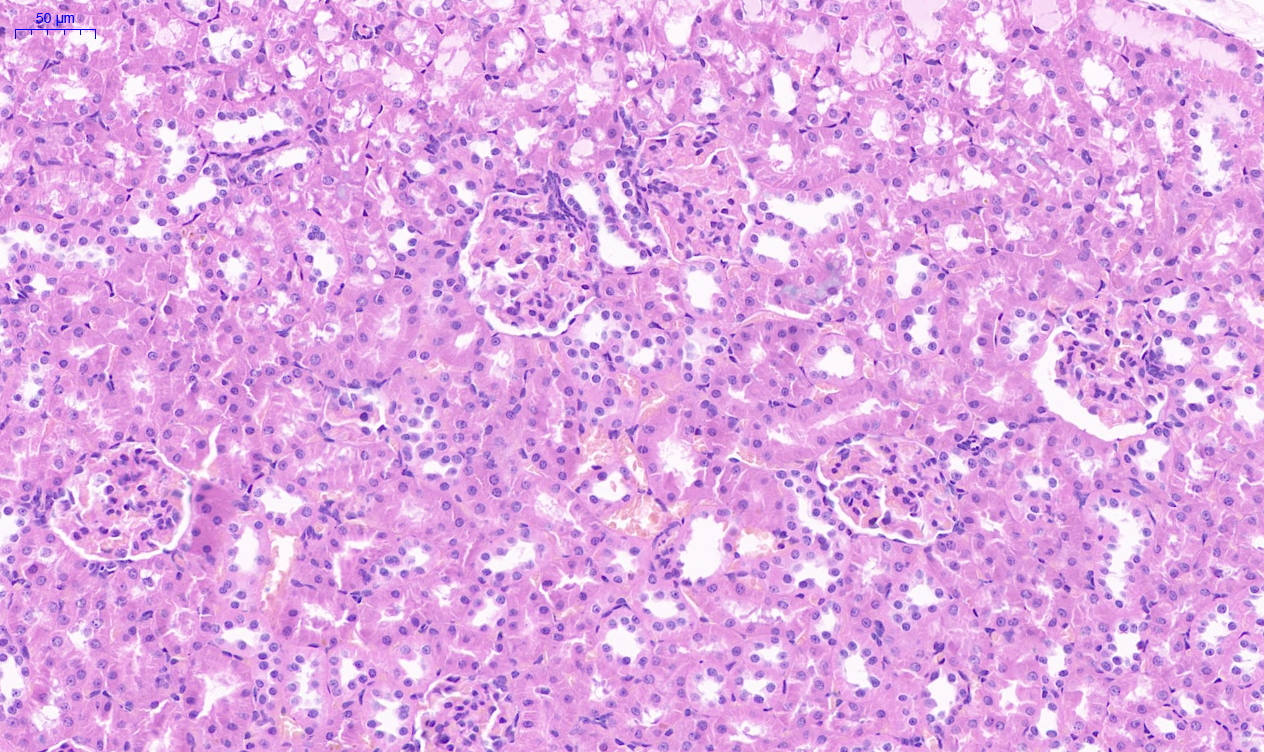

Supplement: Supplemental Information 2 [file peerj-09-12188-s002.zip › The original data-Deng Chen-In English/Fig. 7/8w kidney/normal kidney.jpg]

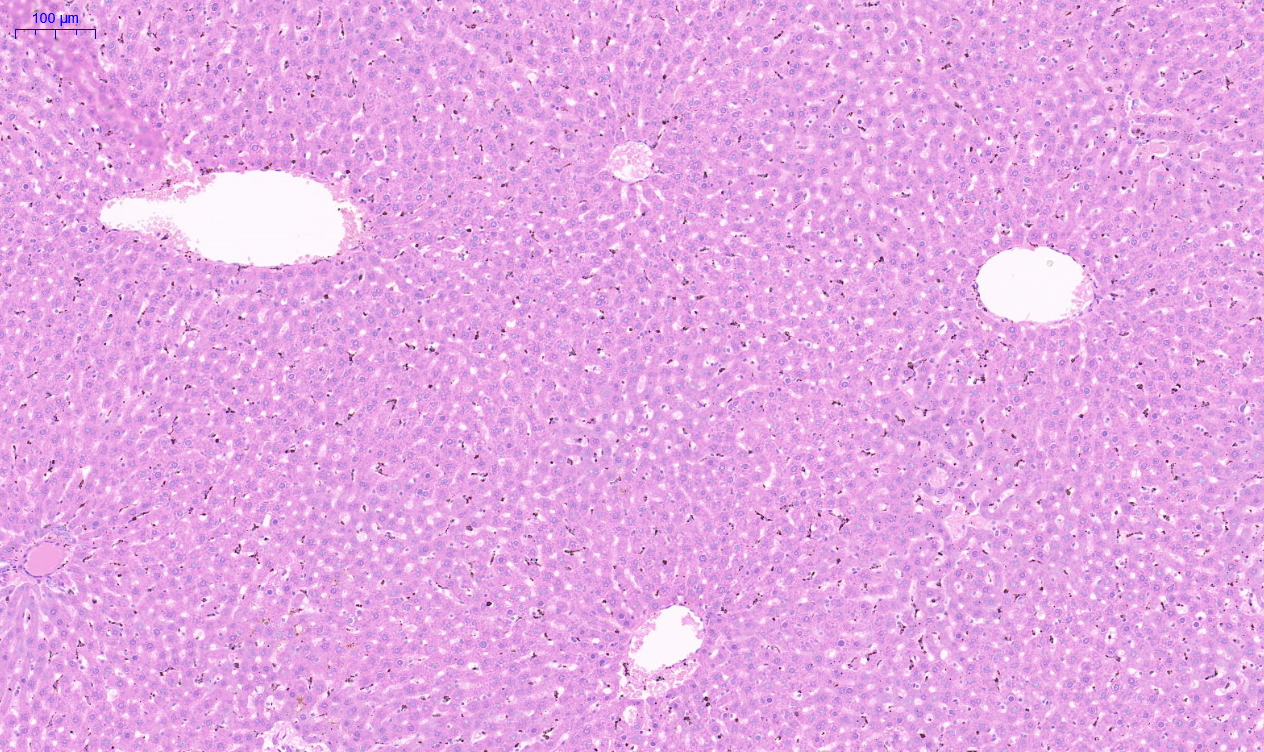

Supplement: Supplemental Information 2 [file peerj-09-12188-s002.zip › The original data-Deng Chen-In English/Fig. 7/8w liver/CS liver.jpg]

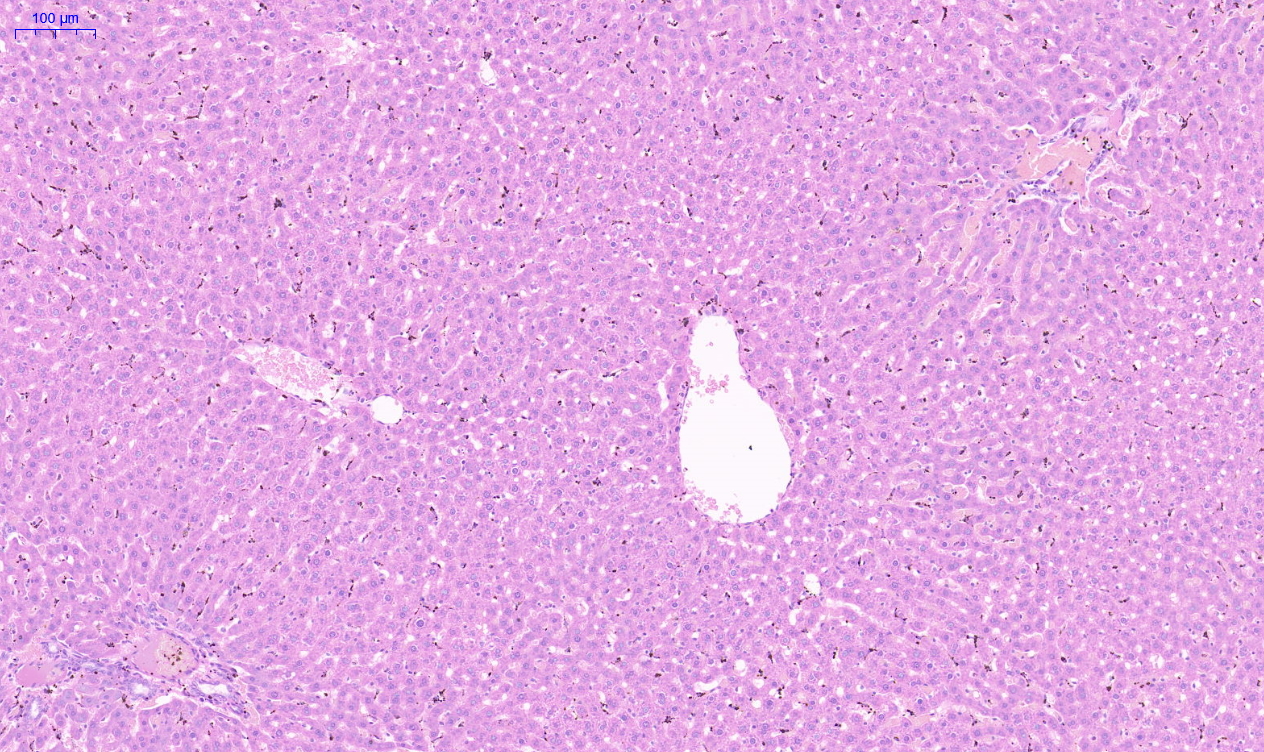

Supplement: Supplemental Information 2 [file peerj-09-12188-s002.zip › The original data-Deng Chen-In English/Fig. 7/8w liver/dECM liver.jpg]

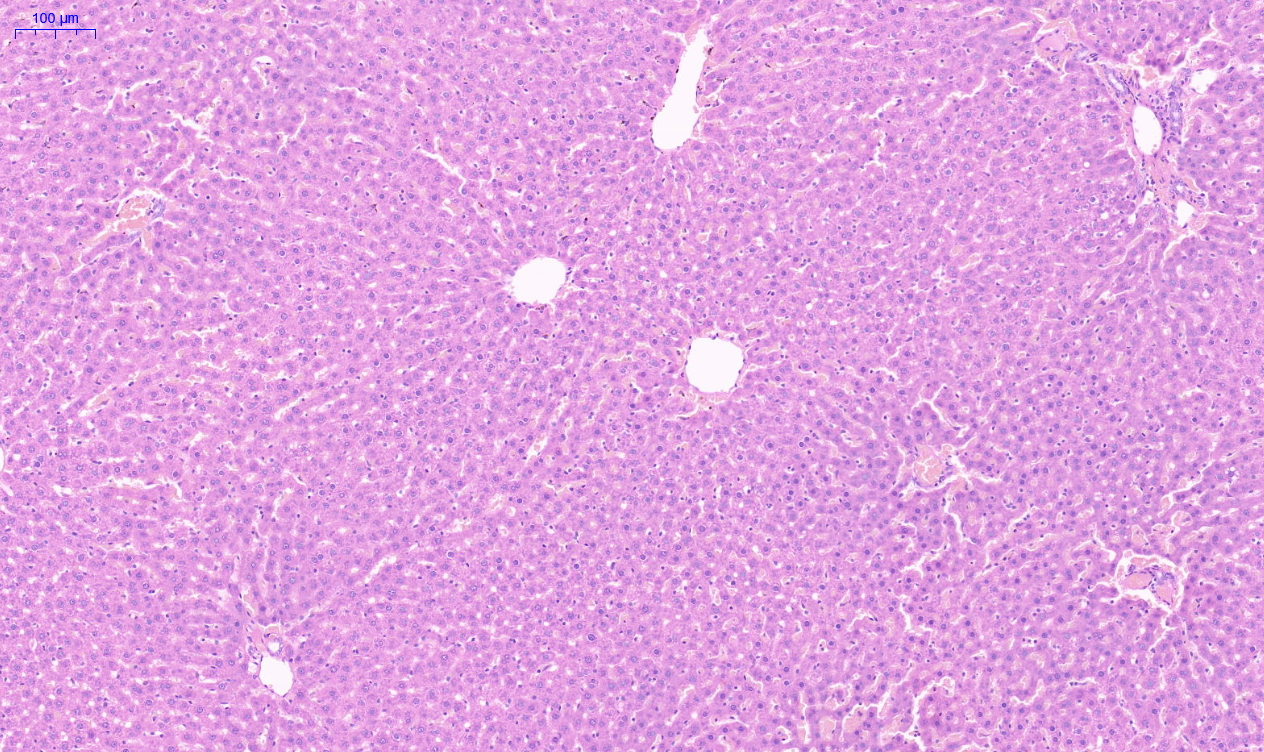

Supplement: Supplemental Information 2 [file peerj-09-12188-s002.zip › The original data-Deng Chen-In English/Fig. 7/8w liver/dECM+CS liver.jpg]

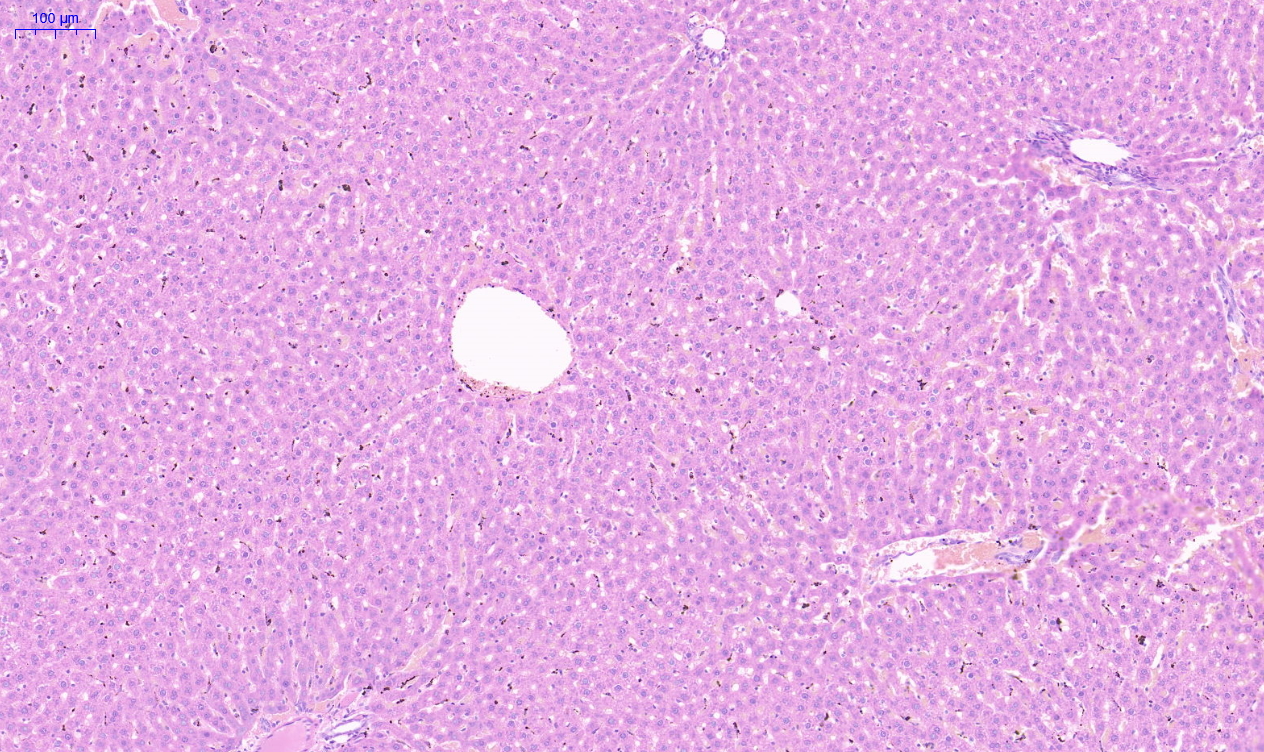

Supplement: Supplemental Information 2 [file peerj-09-12188-s002.zip › The original data-Deng Chen-In English/Fig. 7/8w liver/normal liver.jpg]
